# Alpha-Synuclein PET Tracer Development—An Overview about Current Efforts

**DOI:** 10.3390/ph14090847

**Published:** 2021-08-26

**Authors:** Špela Korat, Natasha Shalina Rajani Bidesi, Federica Bonanno, Adriana Di Nanni, Anh Nguyên Nhât Hoàng, Kristina Herfert, Andreas Maurer, Umberto Maria Battisti, Gregory David Bowden, David Thonon, Daniëlle Vugts, Albert Dirk Windhorst, Matthias Manfred Herth

**Affiliations:** 1 Department of Radiology and Nuclear Medicine, Amsterdam UMC, Vrije Universeit Amsterdam, De Boelelaan 1085c, 1081 HV Amsterdam, The Netherlands; s.korat@amsterdamumc.nl (Š.K.); d.vugts@amsterdamumc.nl (D.V.); ad.windhorst@amsterdamumc.nl (A.D.W.); 2 Department of Drug Design and Pharmacology, University of Copenhagen, Jagtvej 160, 2100 Copenhagen, Denmark; natasha.bidesi@sund.ku.dk (N.S.R.B.); umberto.battisti@sund.ku.dk (U.M.B.); 3 Werner Siemens Imaging Center, Department of Preclinical Imaging and Radiopharmacy, Eberhard Karls University Tübingen, Röntgenweg 15, 72076 Tübingen, Germany; Federica.Bonanno@med.uni-tuebingen.de (F.B.); Adriana.Di-Nanni@med.uni-tuebingen.de (A.D.N.); Kristina.Herfert@med.uni-tuebingen.de (K.H.); Andreas.Maurer@med.uni-tuebingen.de (A.M.); Gregory.Bowden@med.uni-tuebingen.de (G.D.B.); 4 Elysia Raytest, Rue du Sart-Tilman 375, 4031 Liège, Belgium; hoangnguyennhatanh@doct.uliege.be (A.N.N.H.); david.thonon@elysia-raytest.com (D.T.); 5 GIGA Cyclotron Research Centre In Vivo Imaging, Department of Chemistry, University of Liège, Allée du 6 Août 8, 4000 Liège, Belgium; 6 Department of Clinical Physiology, Nuclear Medicine and PET, Rigshospitalet, Blegdamsvej 9, 2100 Copenhagen, Denmark

**Keywords:** alpha-synuclein, imaging, synucleinopathies, positron emission tomography

## Abstract

Neurodegenerative diseases such as Parkinson’s disease (PD) are manifested by inclusion bodies of alpha-synuclein (α-syn) also called α-synucleinopathies. Detection of these inclusions is thus far only possible by histological examination of postmortem brain tissue. The possibility of non-invasively detecting α-syn will therefore provide valuable insights into the disease progression of α-synucleinopathies. In particular, α-syn imaging can quantify changes in monomeric, oligomeric, and fibrillic α-syn over time and improve early diagnosis of various α-synucleinopathies or monitor treatment progress. Positron emission tomography (PET) is a non-invasive in vivo imaging technique that can quantify target expression and drug occupancies when a suitable tracer exists. As such, novel α-syn PET tracers are highly sought after. The development of an α-syn PET tracer faces several challenges. For example, the low abundance of α-syn within the brain necessitates the development of a high-affinity ligand. Moreover, α-syn depositions are, in contrast to amyloid proteins, predominantly localized intracellularly, limiting their accessibility. Furthermore, another challenge is the ligand selectivity over structurally similar amyloids such as amyloid-beta or tau, which are often co-localized with α-syn pathology. The lack of a defined crystal structure of α-syn has also hindered rational drug and tracer design efforts. Our objective for this review is to provide a comprehensive overview of current efforts in the development of selective α-syn PET tracers.

## 1. Introduction

Abnormal alpha-synuclein (α-syn) depositions (α-synucleinopathies) in neurons, nerve fibers, or glia cells are the hallmark for many neurodegenerative diseases such as Parkinson’s disease (PD), dementia with Lewy bodies (DLB), and multiple system atrophy (MSA) [1]. PD, for example, is the most common progressive neurodegenerative movement disorder. Today, approximately 7–10 million patients worldwide are affected. In Europe, the proportion of the people suffering from PD is 1.6% for those aged 65+ and up to 3% for those aged 80+. As the average age of the population in the EU continues to rise, the overall number of PD patients also increases, placing a significant burden on society due to associated healthcare costs [2,3,4]. Currently, the combined direct and indirect cost of PD, including treatment, social security payments, and lost income from an inability to work, is estimated to be nearly EUR 42 billion in the United States of America (USA) alone. Although symptoms can, to a certain extent, be medically remediated, there is currently no cure for PD. Medication costs for an individual with PD average approximately EUR 2100 per year [5].

The underlying biochemical mechanism of PD is not fully understood, and it is difficult to ascertain possible causes of the disease from its effects. More importantly, the clinical diagnosis of PD is not straightforward in the early phases of the illness. This seriously complicates the identification of targets for novel therapies and the interpretation of treatment outcomes. It is imperative to correctly diagnose the illness as early as possible since early intervention is key to limiting the neurodegenerative process. The pathological hallmark of PD is deposition of α-syn protein in the brain followed by dopaminergic neuronal loss [6,7,8]. Currently, α-syn deposition cannot be assessed accurately in vivo. The inability to quantify these abnormal aggregates limits the accurate clinical diagnosis of PD as well as DLB and MSA, and also hinders research into the understanding of these diseases; thus, impeding the development of new treatments. Positron emission tomography (PET) molecular imaging has the potential to reveal the pathogenesis of brain disorders when suitable tracers are available. PET can localize and quantify drug targets, monitor treatment effect, or image disease pathophysiology on a molecular basis. Various studies have indicated toward the importance of imaging with respect to patient inclusion in clinical trials, especially for PD [9]. Currently, imaging of dopaminergic neurons has been used to monitor the effects of investigational disease-modifying drugs in PD, but dopamine cell loss is a downstream effect of α-syn deposition and thus, there is great interest in imaging this specific protein in PD therapies for monitoring progression as well as for early diagnosis. Cerebrospinal fluid biomarkers, skin tissue biopsies, and other imaging techniques will likely be developed in the future as well. However, a PET tracer in the central nervous system (CNS) has the potential to provide evidence for central target engagement, following disease progression and drug treatment efficacy. By developing a tracer that selectively identifies α-syn deposition, the correct cohort of patients may be included in clinical trials and the readout of these trials may be correlated with the underlying disease pathology. Thus, the ability to image α-syn deposition will be a game-changing achievement [7] and will facilitate the development of effective treatments. Presently, there are no tracers available for the detection of α-syn deposition in patients. The main obstacles are difficulties in identifying selective α-syn deposition binding ligands (to be used as tracers) that are suitable for PET measurements. Considering this, the Michael J. Fox Foundation for Parkinson’s Research (MJFF) announced a challenge to develop such a tracer sponsoring a USD 2 million prize to the first successful team.

In this review, we aim to give a detailed summary of the potential radiotracers targeting α-syn developed thus far. For this reason, a bibliographic search was performed from various sources, including PubChem, ChEMBL, Reaxys, World Intellectual Property Organization (WIPO), PubMed and other online resources (in Table S1, Supporting Information). We describe the rationality in their design, highlight lessons to be learned from these efforts, and give suggestions on how to move forward.

## 2. Alpha-Synuclein and PET Imaging

### 2.1. Alpha-Synuclein within the Central Nervous System

α-Syn is an abundant protein throughout the CNS. It represents approximately 1% of cytosolic proteins in the brain and is predominantly found in the neocortex, hippocampus, substantia nigra, thalamus, and cerebellum [10]. Although α-syn has been localized within the nucleus of mammalian neurons [11,12], the vast majority of α-syn has been found in presynaptic termini in a free or membrane-bound form [13]. The normal functional role of α-syn is still not completely understood, nor is its involvement in CNS diseases [14]. However, α-syn has been suggested to play a specific role in synaptic plasticity. For example, a sustained reduction in α-syn expression levels was detected in specific zebra finch brain regions, which were implicated during song acquisition [15]. α-Syn has also been shown to be an inhibitor of phospholipase D2 (PLD2), which indicates that it has a specific function in membrane trafficking [14,16]. Furthermore, there is evidence that α-syn is involved in neurotransmitter release [14] and more importantly in the formation of toxic radical species [17]. Finally, α-syn has also been suggested to be involved in DNA repair processes, including the repair of double-strand breaks [18].

#### Alpha-Synucleinopathies in PD, DLB and MSA

**Figure 1:**
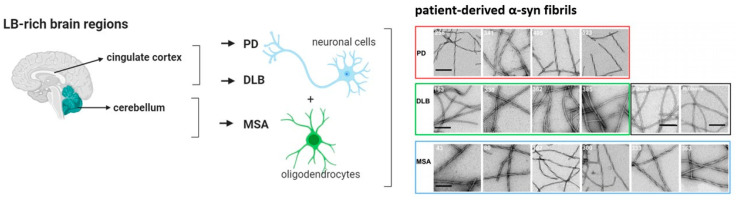
Simplified illustration of the differences of α-syn deposition and structure in PD, DLB, and MSA neurodegenerative diseases. In particular, LB-rich brain regions deposited in the cingulate cortex are thought to be specific for PD and DLB, while depositions within the cerebellum are specific for MSA. Additionally, inclusions of α-syn are found in neuronal cells in PD and DLB. Conversely, in MSA these inclusions are found in oligodendrocytes ((**left**), visualized with BioRender). Structural differences of α-syn have been reported in patient-derived α-syn fibrils (**right**). (Adapted from [24] and visualized with BioRender).

PD patients show abnormal intraneuronal inclusions named Lewy bodies (LB) and Lewy neurites (LN), which are predominantly present in the substantia nigra. Both filament types contain α-syn as a major component. DLB patients also possess LB and LN filaments. In contrast to PD, these filaments are mainly distributed in the cerebral cortex [19]. The major filamentous component of glial and neuronal cytoplasmic inclusions in MSA patients is also α-syn [20]. Contrary to inclusions found in PD and DLB, in MSA, filamentous components are not only found in neurons but also within oligodendrocytes and in the cytoplasm of nerve cells [19,21,22,23] (Figure 1). Patient-derived α-syn fibrils from different α-synucleinopathies, namely PD, DLB, and MSA, possess structural differences. Specifically, PD and MSA fibrils exhibit flat and twisted polymorphs, while, in contrast, DLB fibrils are cylindrical with no twist [24]. The precise role of α-syn in PD, DLB, and MSA is still not understood [25]. However, early α-syn aggregates (protofibrils) and oligomer species that are formed during the aggregation process are believed to mediate neurotoxicity through the disruption of cellular homeostasis and other effects on various intracellular targets leading to mitochondrial toxicity, enhancement of inflammatory responses, and synaptic and endoplasmic dysfunction [26,27,28,29,30,31]. Furthermore, α-syn leads to the release of radical species, which is suggested to trigger neurodegeneration. Finally, DNA repair function also appears to be compromised in LB inclusion bearing neurons [17]. Several overview reviews about the toxic effect of α-syn have been published and summarize the current state-of-the-art knowledge [28,32,33].

### 2.2. The Structure of α-Syn

**Figure 2:**
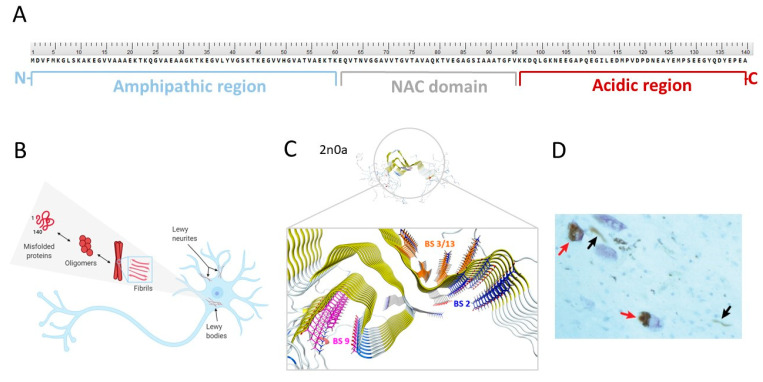
(**A**) Primary structure of α-syn with highlighted regions: N-terminal (in blue), NAC (in grey), and C-terminal (in red). (**B**) Conformational states of α-syn (adapted from [41] and visualized with BioRender). (**C**) Solid-state NMR atomic-resolution (4.8 Å) of α-syn fibrils (PDB ID: 2n0a, adapted from Twohig et al. [42]) with putative binding sites: binding site 2 (BS2; in blue), binding site 9 (BS9; in pink), binding site 3/13 (BS3/13; in orange) on α-syn fibrils (reprinted with permission from [43]. Copyright 2018 American Chemical Society. Structure of α-syn was imported from PDB and visualized with MOE 2018). (**D**) The α-syn pathology of PD with two pigmented nerve cells containing LB (red arrows) and LN (black arrows). (Adapted from [19]).

The native form: Considerable effort has been made to reveal the native state of α-syn. However, it is still debated if native α-syn exists as an unfolded monomer [34], as a helically folded tetramer [35], as multimer conformations [36], or as a mixture of all these forms [36]. In contrast, the primary structure of α-syn is well described. It is encoded by the SNCA gene and encompasses 140 amino acids (Figure 2A). The protein is divided into three domains: the N-terminal amphipathic region (1–60 residues), the non-amyloid-β component (NAC) hydrophobic region (61–95 residues), and the C-terminal acidic region (96–140 residues) [37]. Several isoforms of α-syn exist and are formed through alternative splicing [38,39]. The synuclein protein family is reported to undergo extensive post-translational modifications (PTMs) in vivo including phosphorylation, truncation, acetylation, or nitration [40].

α-Syn fibril formation: Under certain physiological conditions, native α-syn can aggregate. Thus far, it is unclear if a specific conformation of α-syn or other processes trigger aggregation and ultimately, convert soluble native α-syn into β-sheet rich, tightly stacked fibrils. However, aggregation is thought to occur via a highly dynamic equilibrium between numerous conformations, various oligomeric states and (proto)fibrils (Figure 2B) [36]. Understanding the aggregation process is difficult since it is highly complex and can be affected by a broad set of parameters. For instance, various environmental factors appear to have an influence on an increased risk for PD, such as exposure to pesticides, herbicides, exposure to heavy metals, organic solvents, and various sources of oxidative stress [44]. Conversely, it has also been shown that certain mutations within the α-syn encoding SNCA gene enhance or reduce the formation of protofibrils [45]. Changes within the α-syn NAC domain appear pivotal for α-syn fibrillation, especially within the amino acid sequences ^71^VTGVTAVAQKTV^82^ or ^66^VGGAVVTGV^74^. Removal of either of these sequences abrogated the fibril formation completely [46,47]. PTMs have also been shown to influence the aggregation process. For example, phosphorylation on residue S129 leads to extensive aggregation (Figure 2D) [48]. This PTM appears to be pathologically relevant since it is present in >90% of all α-syn derived from PD patients, but only seen in 4% of native soluble α-syn [49].

Three-dimensional (3D) structure and possible binding sites for small molecules: To date, no high-resolution and detailed 3D structure of α-syn fibrils has been reported. The highly repetitive nature of the respective secondary structure and the residue-type degeneracy pose significant challenges to this endeavor [50]. The lack of knowledge of the function of α-syn, its binding partners, their binding mechanism, and the broad set of possible PTMs have been found to be additional complications [37]. Nevertheless, certain structural secrets of α-syn fibrils have been revealed [50,51,52]. For example, Tuttle et al. could have elucidated the structure of a single filament fibril of α-syn using solid-state nuclear magnetic resonance (ssNMR) (Figure 2C) [50]. Years later, Li and colleagues were the first to distinguish between two structurally different forms of fibril species, termed protofilaments, using cryogenic electron microscopy [53]. In 2018, Hsieh et al. conducted a molecular blind docking study using the structural data obtained from the ssNMR study by Tuttle et al. This study aimed to identify possible binding regions of small molecules toward α-syn fibrils [43]. Three putative binding sites, namely binding site 2 (Y39-S42-T44; BS2), 9 (G86-F94-K96; BS9), and 3/13 (K45-V48-H50 and K43-K45-V48-H50; BS3/13) were identified (Figure 2C). To test these virtual hits, chemically diverse putative binders were synthesized, and their binding profile was examined using photoaffinity labeling and radioligand binding studies. Results strongly indicated that there are several binding sites [43]. How this knowledge can be used to design selective and high-affinity α-syn ligands has to be further investigated.

### 2.3. Challenges—Why Is It Difficult to Develop an α-Syn PET Tracer?

Challenge 1. The target

**Figure 3:**
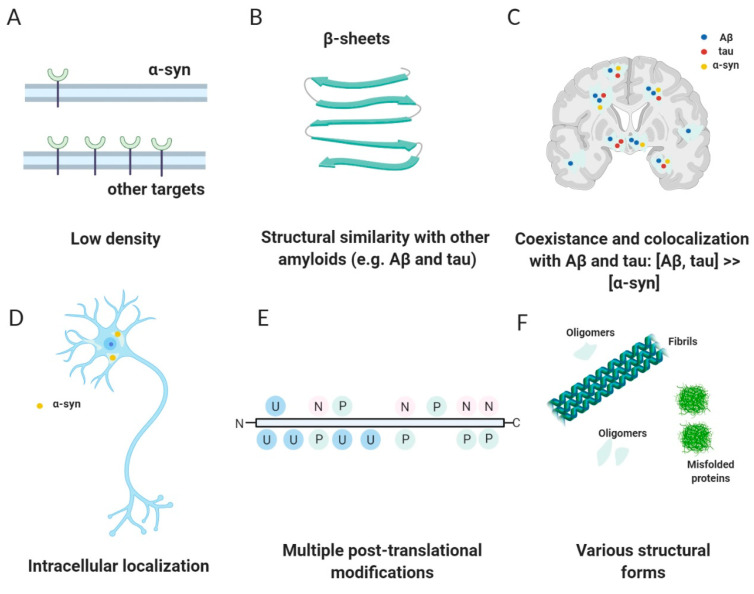
Challenges and characteristics of an ideal α-syn tracer. (Reprinted with permission from [56]. Copyright 2014 the Society of Nuclear Medicine and Molecular Imaging, Inc.) (**A**) Low density and concentration of α-syn in comparison with other targets. (**B**) Structural similarity with other proteins such as Aβ and tau. (**C**) Coexistence and colocalization with structural similar amyloids such as Aβ and tau. (**D**) Intracellular localization. (**E**) Multiple post-translational modifications such as phosphorylation (P), ubiquitination (U), and nitration (N). (**F**) Various structural forms such as fibrils, oligomers, and misfolded proteins. (Visualized with BioRender).

The development of an α-syn PET tracer is more challenging in comparison to the development of tracers for other amyloids such as Aβ or tau. A key reason for this is that the absolute concentration of α-syn aggregates within the brain is thought to be 10- to 50-fold lower than that of Aβ or tau (Figure 3A) [7,54,55]. Consequently, high affinity of a putative tracer, most likely in the subnanomolar range, is required. Next, co-existence and co-localization of α-syn with Aβ and tau fibrils requires a PET tracer with high selectivity over Aβ and tau in order to be able to image α-syn at all (Figure 3C) [56]. For instance, Aβ pathology can be found in more than 80% of all synucleinopathies. In DLB, Aβ deposits are detected in approximately 85% of all cases and tau pathology is observed in approximately 30% [7,54,57,58]. Unfortunately, Aβ or tau fibrils form structurally similar β-pleated sheets (Figure 3B), complicating the development of selective α-syn binding ligands. Furthermore, and in contrast to Aβ, the highest concentration of α-syn inclusions can be found intracellularly (Figure 3D). Therefore, any successful α-syn PET tracer has to be able to cross cell membranes, in addition to the blood-brain-barrier (BBB) [54,56]. Usually, this is not considered the biggest hurdle, but it may lead to issues, e.g., the D_2/3_ PET tracer [^11^C]raclopride can pass the BBB, but it cannot pass cell membranes [59]. It might also be that extensive in vivo PTMs possibly result in a variety of substructures that might not be recognized by the tracer developed for the unmodified structure (Figure 3E) [54,56].

Challenge 2. The design

Another aspect that in general challenges the development of tracers for amyloids is the standard development strategies that have been established over the last decades for more traditional targets such as receptors and enzymes that cannot be applied to these protein-based structures without precisely defined binding pockets. The underling binding mechanism is poorly understood and studies identifying for example binding sites, or to reveal kinetic and thermodynamic ligand-binding parameter using computational or other strategies are in its infancy [43,60,61]. Conversely, structure-based drug design of these ligands is hampered by the limited availability of high-resolution X-ray crystal structures of α-syn fibrils. Moreover, it is questionable if these structures are able to mimic the aggregates and conditions of the fibrils in vivo. For this reason, the only option available to develop an α-syn PET tracer is a ligand-based approach. However, this strategy has several limitations mainly related to the small number of ligands and data available for the binding. Furthermore, the extensive literature and structures available on Aβ or tau tracers cannot be exploited to design new compounds in order to avoid selectivity issues.

Challenge 3. The evaluation

The reliability and predictability of in vitro binding assays is crucial in any PET tracer development and is a big challenge for α-syn tracer development. It has been postulated that structural variation of α-syn between patient brain-derived in vivo aggregates and in vitro α-syn fibrils can explain the difficulties in translating new α-syn PET tracers to clinical research [62]. Structural heterogeneity between in vivo brain-derived material from PD and MSA patients have been reported and support this hypothesis [62,63]. Do these conformational differences have an influence on the binding pattern of potential ligands that have still to be elucidated, i.e., are binding motifs in different conformational α-syn fibrils changed in such a way that prevent ligands to bind universally to all forms (Figure 3F)?

Similar considerations also apply to α-syn depositions formed in animal models [7]. Are these forms structurally related to those formed in humans and can they be used for screening purposes to identify selective ligands? In this context the selection of the animal model plays a crucial role in the outcome of these experiments. In general, in vivo animal testing for tracers targeting brain aggregates is currently performed in wild-type healthy animals such as mice, rats, and non-human primates (NHP). However, recent studies are moving toward the use of disease models since they are able to mimic intracellular inclusion bodies that are as similar to those found in humans. Currently, several genetically modified rodent models are available. These are usually robust, but the long time required for the development of the pathology (several months) and the high costs limit the use of these animals. For these reasons, the most employed rodent models are the seeding model with pre-formed fibrils (PFF), viral vector model, and the combination of viral vectors and PFF [64,65,66]. The combination of a viral vector and PFFs have shown all the important hallmarks of α-synucleinopathies and was reported to accelerate the induction of α-syn aggregates as rapid as within 10 days after PFF injection [65] Another quick, robust, and easy to implement model was developed by Verdurand et al. by injecting fibrils into striatum of rats and conducting in vivo imaging 7 days after inoculation [67]. Rodents may not provide all the data (safety, P-gP efflux, metabolites, etc.) for the tracer, and they need to be tested in larger animals. NHP models with α-syn inclusion have been created with inoculation of fibrils, LB, and viral vectors but all the models require months to form the pathology, making them expensive [68,69,70]. The optimum would be to develop a rodent model of α-synucleinopathy that is suitable for in vivo PET imaging of α-syn pathological forms. The model must fulfil the criteria of rapid, abundant, and progressive pathology development over time. Another animal that has gained popularity in brain imaging is the domestic pig [71,72]. Creating an α-syn pig model that is appropriate, fast, and inexpensive will drastically reduce the cost of developing such tracers.

It is doubtful if in vitro assays or in vivo models based on fibrils are predictive and clinically relevant; models to study specific uptake in vivo are lacking. For this reason, exclusively patient-derived material should be used for screening new α-syn tracers. However, access to human tissue that is well characterized in respect to its pathology is limited and application of high-throughput screening methods to this material is currently not available. This obviously slows down the development process.

### 2.4. Success Criteria for a Small Molecule α-Syn PET Tracer

**Figure 4:**
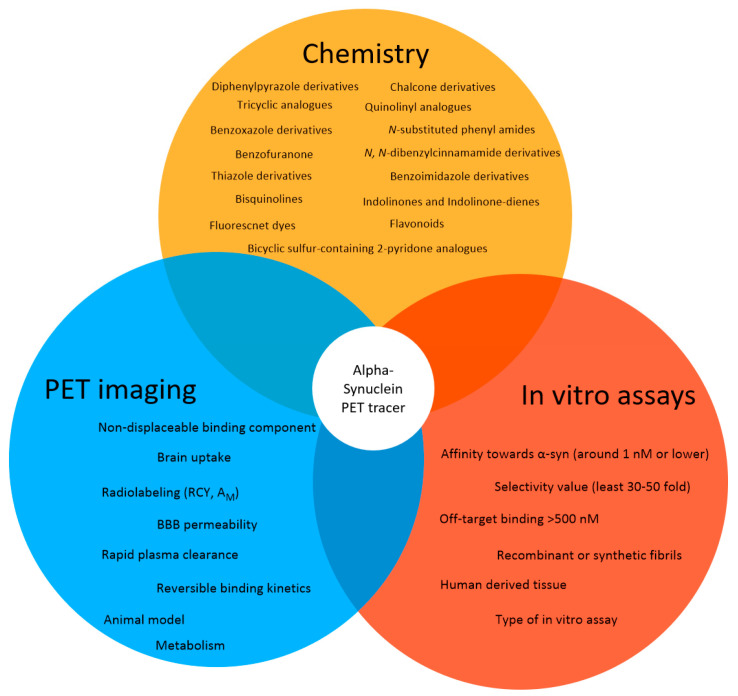
The approach for the development of α-syn selective PET tracers. The initial search was conducted from various sources (in Table S1, Supporting Information) in order to create an overview of compounds that have been designed and developed to image α-syn. The identified structures should be screened using criteria similar to the structures proposed by the MJFF for a successful α-syn PET tracer and are based on in vitro assays and PET imaging technique.

A successful PET tracer for neuroimaging is dependent on several key parameters (Figure 4) [73]. First, the ligand must be labeled in sufficient amounts (activity yield >500 MBq). Usually, carbon-11 (^11^C) or fluorine-18 (^18^F) are the nuclides of choice for small molecule-based PET neuroimaging tracers. These nuclides display excellent decay characteristics for PET imaging (short half-life, good branching ratio, and short positron range) [74] and radiotracers labeled using these radionuclides can also be produced in good radiochemical yields (RCY) in most well-equipped radiopharmacies (activity yield >500 MBq) [75]. Additionally, potential ligands can be radiolabeled with these isotopes without introducing bulk and charge, which often prevents sufficient blood-brain barrier (BBB) penetrance. BBB permeability should be high with an early standard uptake value (SUV) >1.0 [54] (within several minutes, for example 5-10 min). Brain uptake for amyloid tracers is recommended to be ≥ 0.4% ID/g in rat brain or ≥ 4.0%ID/g in mouse brain [54]. Additionally, the plasma clearance half-time should be fast (less than 30 min) [54] to reduce background signal from blood. Ideally, the tracer binds reversibly to its target and no metabolites exist within the brain. These characteristics greatly simplify kinetic modeling and allow for appropriate quantification and reproducibility [56]. The tracer should show a low non-displaceable binding component to increase signal-to-noise ratio within the brain [54] and has to be accessible in high molar activity (A_m_) (>50 GBq/µmol, we believe) to avoid target saturation or non-negligible self-blocking [76,77]. An additional key parameter for any successful neuro PET tracer is its selectivity toward off-targets expressed or deposited in same brain regions. A recommended selectivity over these off-targets is thought to be > 30–100 [78]. For a successful α-syn PET tracer, the MJFF has published a criteria list, which has to be fulfilled to receive the MJFF Alpha-synuclein Imaging Prize [79]. We have adapted this list and will use it to assess the ability of published structures to image α-syn via PET (Figure 4).

#### Suggested Success Criteria for a α-Syn PET Ligand in the Preclinic Setting

- Radiolabeling with ^11^C or ^18^F should be achievable in a RCY of >10% (activity yield >500 MBq) and in a high molar activity (>50 GBq/µmol);
- Affinity toward α-syn around 1 nM or lower;
- The radioligand enables quantification of lower density α-syn targets in the presence of higher density Aβ or tau proteins;
- The binding affinity or density selectivity for α-syn over Aβ or tau should be at least 30–50 fold (~1 nM vs. 50 nM);
- Off-target binding > 500 nM;
- Selective binding to α-synuclein-rich brain homogenates from PD patients (versus Aβ, tau rich homogenates);
- Binds to LBs/LNs in human tissue;
- BBB permeability, with early peak uptake of SUV >1.5;
- ≥0.4% ID/g in rat brain or ≥4.0% ID/g in mouse brain;
- No or minimal radiometabolites within the brain;
- Low non-displaceable binding component (rule of thumb: logD_7.4_ < 3 [80];
- Reversible binding kinetics with rapid plasma clearance (<30 min).

## 3. Alpha-Synuclein PET Tracer Development—From a Molecular Development Point of View

Over the past decades, a set of chemically diverse compounds have been developed that target α-syn. The development was based on various strategies including rational drug design and high-throughput screening approaches. In this section, we will present a selection of these structures and highlight their potential to be used as α-syn selective PET tracers. Additionally, we will critically reflect their in vitro and in vivo biological evaluation results.

**Figure 5:**
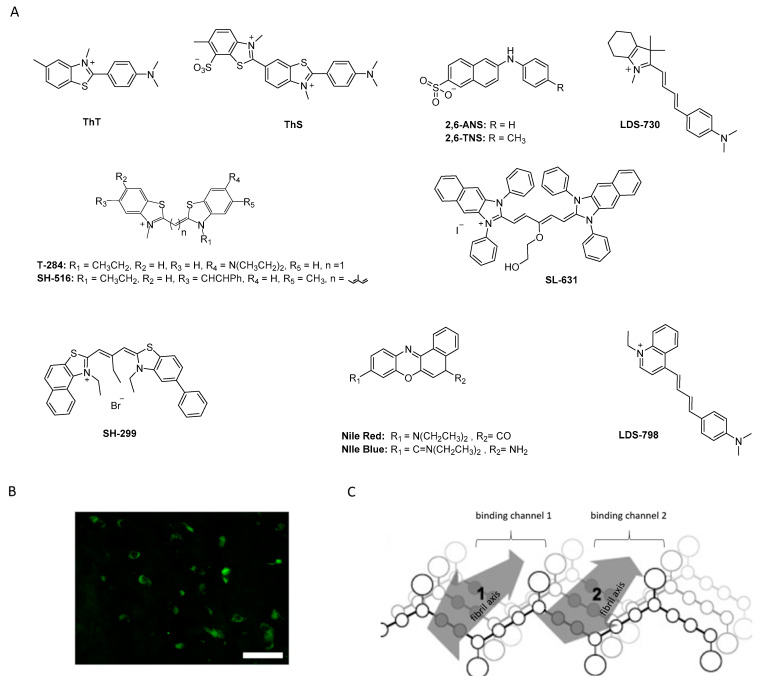
(**A**) Chemical structures of fluorescent probes and cyanine dyes. (**B**) ThS positive neurons confirming α-syn pathology (adapted from Patterson et al.) [88]. (**C**) Hypothetical presentation of how mono- and trimethine cyanine dyes interact with α-syn fibrils (reprinted with permission from [83]. Copyright 2008 Elsevier).

Historically, the first investigated α-syn binders were based on fluorescent dyes such as Thioflavin-T (**ThT**) or Thioflavin-S (**ThS**) (Figure 5A,B) [81]. These structures, however, were not selective and consequently, numerous attempts to develop fluorescent dyes with an improved binding affinity have been made over the years. For example, Celej and colleagues studied the effect of N-arylaminonaphthalene sulfonate derivatives upon interaction with conjugated α-syn fibrils. **2,6-ANS** and **2,6-TN** (Figure 5A) showed little fluorescence in the presence of non-aggregated α-syn, but much more fluorescence, occasionally with spectral shifts, when applied to fibrils [82]. Affinities were, however, only in the low micromolar range. In the same year, Volkova et al. conducted a similar study with monomethine cyanines. **T-284** and **SH-516** were shown to associate with fibrillar α-syn with affinities of 560 nM and 650 nM, respectively (Figure 5A) [81,83]. To better understand the interaction of these structures, especially **SH-516** was studied in a model proposed by Krebs et al. that describes the binding of dyes to amyloids [84]. It was suggested that monomethine cyanines bind in a long channel that run parallel to the length of the fibril axis (Figure 5C) [85]. This information was used to develop second generation dyes. The resulting tri- and pentamethine cyanines displayed selectivity toward fibrillar or oligomeric α-syn over monomeric α-syn [83]. Interestingly, the pentamethine cyanine dye **SL-631** (Figure 5A) also bound better to less dense and structured oligomers compared to beta-sheet containing fibrils. In contrast, the trimethine cyanine **SH-299** (Figure 5A) was able to bind to β-sheets. This distinct binding pattern was attributed to the distinct steric characteristics of both compounds. The more sterically hindered structure **SL-631** is supposed to be incapable of entering and binding in the groove formed by β-sheets, whereas the less bulky structure **SH-299** is thought to be capable. **SH-299** may serve as an imaging agent for both oligomeric and fibrillar α-syn species whereas **SL-631** is better suited for oligomeric forms [86]. In 2013, Neal et al. developed three heterogeneous structures to image α-syn selectively. **LDS-798** and the phenoxazine dyes **Nile Red** and **Nile Blue** (Figure 5A) were able to selectively bind to LB in postmortem human tissue of DLB or PD patients. Selectivity over amyloid or tau pathology in human AD tissue was detected. Surprisingly, to date no further attempts concerning radiolabeling and binding affinity measurements have been conducted [87].

From a broader point of view, the studies mentioned above show that it is possible to selectively bind and image α-syn in postmortem human tissue. However, most probes displayed low affinity and poor selectivity toward α-syn and are, as such, not suitable for development as PET tracers. Conversely, these ligands were considered excellent lead structures and were consequently employed as a starting point to develop other α-syn selective ligands in many of the studies discussed below.

### 3.1. Tricyclic Analogues (TCAs)

#### 3.1.1. Phenothiazine Analogues

**Figure 6:**
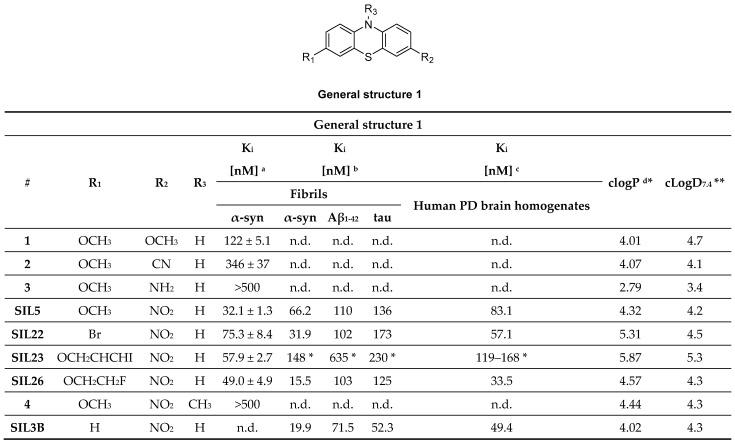
Phenothiazine analogues with binding properties determined from two different assays. ^a^ Affinity toward α-syn fibrils determined by ThT fluorescence assay [92]. ^b^ Affinity toward recombinant α-syn and tau and synthetic Aβ_1–42_ fibrils determined by [^125^I]SIL23 competition assay [55]. ^c^ Affinity toward human PD brain homogenate determined by [^125^I]SIL23 competition assay [55]. * K_d_ value determined with the radioligand [^125^I]SIL23 on fibrils or human PD brain homogenate. * clogP computed from BioByte software. ** clogD_7.4_ computed from ACD/Percepta. n.d.: not determined.

Phenothiazine analogues (SILs) have widely been studied as potentially selective α-syn PET ligands. They were developed based on rational drug design considerations. For example, structurally related bisarylimines have been proven to show neuroprotective effects in PD related models, most likely due to their antioxidant properties [89]. Within this structural class, several phenothiazines were found to inhibit insoluble α-syn filament aggregation at low micromolar IC_50_ concentrations [89,90,91]. Encouraged by these findings, several phenothiazines analogues were synthesized and their binding properties evaluated based on a fluorescent ThT competition assay. Several ligands with affinities between 30 and 80 nM were identified (Figure 6). The first promising structure identified within this study was a dimethoxy substituted phenothiazine analogue with an affinity of approximately 120 nM. Replacement of one of the methoxy group with a cyano-group or an amine decreased the affinity more than 3 to 5-fold. In contrast, replacement with a nitro-group increased affinity approximately 4-fold (**SIL5**, Figure 6) [92]. Further attempts to improve the affinity by altering this position with other substituents failed in a follow-up study [93]. Structure activity relationship (SAR) studies around the methoxy group of **SIL5** (K_i_ between 32.1 nM and 83.3 nM, Figure 6) revealed that the binding affinity toward α-syn decreased in the following order: –OCH_3_ > –Br > –I > –H. However, the fluoroethoxy (**SIL26**) and the (3-iodoallyl)oxy (**SIL23**) derivatives showed only slightly reduced affinity in the order of 50 nM [92]. Furthermore, *N*-substitution of phenothiazines greatly reduced affinity against α-syn fibrils [92,94]. In 2018, binding of SILs toward α-syn fibrils was studied. In particular, **SIL23** and **SIL26** preferentially bind to BS 3/13 of α-syn fibrils [43].

**Figure 7:**
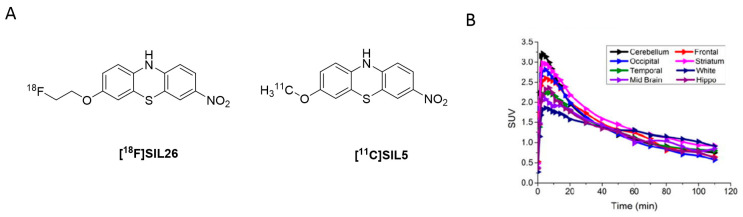
Radiolabeled phenothiazine analogues. (**A**) Chemical structures of **[^18^F]SIL26** and **[^11^C]SIL5**. (**B**) TACs from in vivo evaluation of **[^11^C]SIL5** in healthy NHP (adapted from) [95].

Inspired by these findings, Bagchi et al. radiolabeled **SIL23** with iodine-125 and evaluated its selectivity profile against Aβ_1–42_ and tau fibrils as well as its binding properties at LBs and LNs present in human PD brains. In this study, **SIL23** showed a K_d_ value of 148 nM on α-syn fibrils and comparable affinity in human tissue. These values are, however, a 2-fold lower than the affinity determined by the fluorescent ThT competition assay, highlighting the variations between both assays. Its selectivity toward Aβ_1–42_ and tau fibrils is low and as such **SIL23** is not suitable for the development of a PET α-syn tracer. However, **[^125^I]SIL23** is, nonetheless, an excellent tool to evaluate the binding profile of other phenothiazine analogues. **SIL26** showed the best selectivity profile in this assay. A 6-fold and 8-fold selectivity for α-syn over Aβ_1–42_ and tau fibrils was detected, respectively. In general, K_i_ values obtained from PD brain homogenate assay were comparable with those obtained from α-syn fibrils. This indicated that not only can the **[^125^I]SIL23** competition assay accurately predict binding in human tissue, but also that the fibrillar α-syn binding site is conserved between recombinant α-syn fibrils and the in vivo formed species in PD patients [55]. Although **SIL5** and **SIL26** did not show particularly high binding affinity and selectivity, they showed most promising results among the tested phenothiazines and were therefore radiolabeled for in vivo PET studies (Figure 7A). **[^11^C]SIL5** was isolated in a RCY of 35–45% (decay-corrected to EOB) and an A_m_ of >363 GBq/µmol and **[^18^F]SIL26** in a RCY of 55–65% (decay-corrected to EOB) and an A_m_ of > 200 GBq/µmol [95]. Both tracers were able to pass the BBB and had homogeneous distribution throughout the brain in male Sprague-Dawley rats. Total brain uptake for **[^11^C]SIL5** was 0.953%ID/g at 5 min p.i. and 0.287%ID/g at 30 min p.i. In contrast, **[^18^F]SIL26** showed a lower total brain uptake of 0.758%ID/g at 5 min p.i. but a higher uptake than **[^11^C]SIL5** (0.359%ID/g) at 120 min p.i. The decreased washout may be a result of the increased lipophilicity of **[^18^F]SIL26** in comparison with **[^11^C]SIL5**. Faster washout kinetics are generally preferred in PET studies, [96] and consequently, **[^11^C]SIL5** was selected for in vivo PET imaging studies in healthy NHP (Figure 7B). Expected homogenous uptake within the brain was observed (since no target should be present in healthy subjects) [95]. Standardized uptake value (SUV) peak uptake was > 3 at approximately 7 min and slowly decreased and flattened over 70 min to values around 1 SUV. This value is a good indicator for the non-displaceable binding component of the tracer and may be too high, taking into account the low amount of α-syn fibrils that should be present in diseased animals. As such, a further study should be performed in α-syn positive animals, e.g. transgenic mice expressing α-syn fibrils, to see if **[^11^C]SIL5** can selectively image α-syn. It has been shown that, at least for **SIL23,** the binding site in mice expressing the human α-syn transgene containing the A53T mutation (M83 line) is present in this transgenic mouse model for PD, whereas mice expressing the WT human α-syn transgene (M7 line) did not show any specific **SIL23** in brain tissue homogenates. Since **SIL5** is a close analogue of **SIL23** and can therefore be displaced by **SIL23**, it is likely that both compounds bind at the same binding site. As such, the A53T transgenic mouse model appears to be a suitable in vivo model to test the in vivo binding characteristics of **[^11^C]SIL5** or related compounds [95].

#### 3.1.2. Phenoxazine and Phenazine Analogues

**Figure 8:**
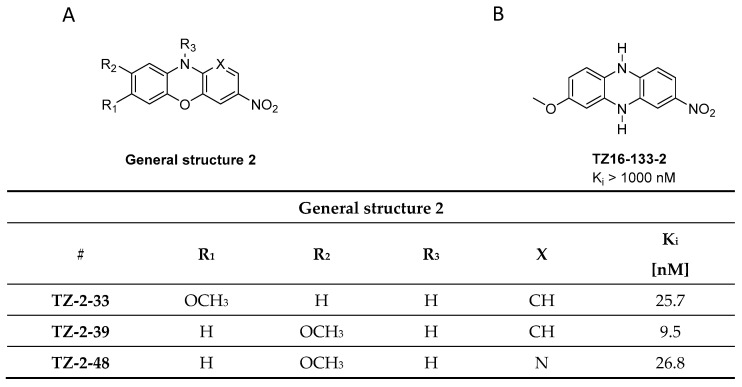
General structure of phenoxazine and phenazine analogues. (**A**) Chemical structure of phenoxazine analogues with binding properties utilized from ThT fluorescence assay using α-syn fibrils [97]. (**B**) Chemical structure of phenazine analogues **TZ16-133-2** with binding affinity K_i_ > 1000 nM [97].

In parallel to the phenothiazines, their isosteres, the phenoxazines and the phenazines, were also evaluated as potential α-syn PET tracers. Tu et al. synthesized a small compound library consisting of 8 compounds and determined their binding affinity toward recombinant α-syn fibrils by a ThT-based fluorescence assay. Several ligands showed affinity in the nanomolar range (Figure 8A) and among them **TZ-2-39** with the lowest K_i_ value of 9.5 nM [97]. In comparison with the phenothiazines, the phenoxazines showed better affinity toward α-syn fibrils. For example, **SIL5** showed a K_i_ of 32.1 nM while **TZ-2-33** a K_i_ of 25.7 nM. Values were determined using the same binding assay set-up. No further attempts have been made thus far to improve the binding profile of this structural class. **TZ-2-48** showed displaceable binding and was shown to bind preferentially to the BS 3/13 position of α-syn fibrils [43]. Interestingly, the phenazines (Figure 8B) did not show any significant affinity toward α-syn, highlighting the importance of oxygen or sulfur in the bridge between the aromatic rings [97].

### 3.2. Benzoxazole Derivatives

#### 3.2.1. BF-227

**Figure 9:**
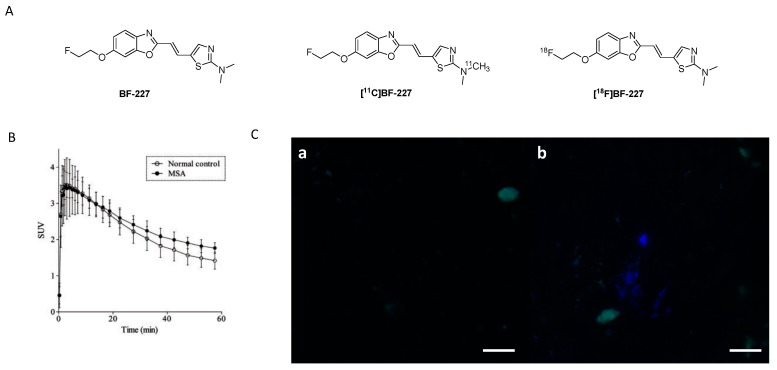
(**A**) Chemical structures of **BF-227**, **[^11^C]BF-227** and **[^18^F]BF-227**. (**B**) TACs of **[^11^C]BF-227** in putamen in normal controls and MSA patients (Reprinted with permission from [100]. Copyright 2010 Oxford University Press). (**C**) Fluorescence detection of BF-227 (at 100 µM) using MSA brain sections. (**a**) Tissue autofluorescence (in green) that is imaged prior to BF-227. (**b**) BF-227 labeling (in blue) of few inclusions. Microscopic examination under DAPI (blue, exposure 500 ms) and FITC (green, exposure 100 ms) filters (adapted from [101]).

**BF-227** was initially developed as a PET ligand to image amyloid plaques in AD patients (Figure 9A) [98]. However, during its biological evaluation **BF-227** was found to bind also to α-syn fibrils with an affinity of K_d_ 9.63 nM, 10-fold lower than its affinity to Aβ_1–42_ fibrils (K_d_ 1.31 nM). Inspired by these results, **BF-227** was evaluated in human brain tissue. Contradictory results with respect to the ability of **BF-227** to detect α-syn in human brain tissue have been reported [99,100,101,102]. For example, Fodero-Tavoletti and colleagues reported that **BF-227** binds to α-syn in PD, but not in DLB patients [99]. A follow-up study analyzing the binding properties of **BF-227** in human postmortem tissue using brain specimens from PD and DLB patients showed that **BF-227** can detect α-syn fibrils in all cases [99]. A PET study in MSA patients confirmed that **[^11^C]BF-227** (Figure 9A,B) is suitable for imagingα-syn deposition in these patients [100], but a recent publication by Verdurand et al. questioned the binding of **BF-227** to pathological forms of α-syn. Autoradiography (ARG) on MSA and control brain sections without α-syn pathology were investigated. In contrast to previous studies, Verdurand and colleagues selected the medulla oblongata as their target region to study the binding of **BF-227**. The reason for this is that the medulla oblongata is systematically affected during disease progression in MSA without developing Aβ co-pathology. **[^18^F]BF-227** (which is chemically identical to **[^11^C]BF-227**) could not detect any α-syn inclusions when compared to healthy controls [101]. This study concluded that **BF-227** cannot be used as an α-syn PET ligand [99,100]. The discrepancy between results was explained with concentration differences of **BF-227** used in ARG and fluorescent measurements. In contrast to fluorescence measurements, where micromolar concentrations are required, only nanomolar concentrations are needed in ARG. The concentration differences stem from the sensitivity of fluorescence compared radioactivity detection. Authors exemplified the latter by conducting fluorescence study and exposing MSA patient at 100 µM of unlabeled **BF-227** without corresponding ARG detection at 1 nM (Figure 9C) [101]. However, the given explanation cannot explain the positive results gained within the PET study of **[^11^C]BF-227** [100]. Verdurand and colleagues suggest that PET differences in this study are unlikely related to α-syn binding. More importantly, they showed that **BF-227** can selectively bind to Aβ and as such suggesting that mixed Aβ or α-syn pathologies, which often coexist, could have been imaged in the aforementioned PET study [101].

These findings highlight the difficulties of validating the binding properties of a given chemical structure for its suitability for imaging α-syn fibrils. Control experiments are of crucial importance to rule out binding to other fibrillar structures such as Aβ_1–42_, especially in human tissue. Evaluation assays should be carefully validated with respect to their ability to predict whether a chemical structure is able to image α-syn fibrils in human tissue. Different conformational forms and densities of α-syn in MSA, DLB, and PD tissue can also influence the outcome of a study and lead to false conclusions. In summary, further experiments are required to validate the usefulness of **BF-227** as an α-syn PET ligand. Current results suggest that **BF-227** is not capable of imaging α-syn fibrils in vivo.

#### 3.2.2. SAR Studies of BF-227

**Figure 10:**
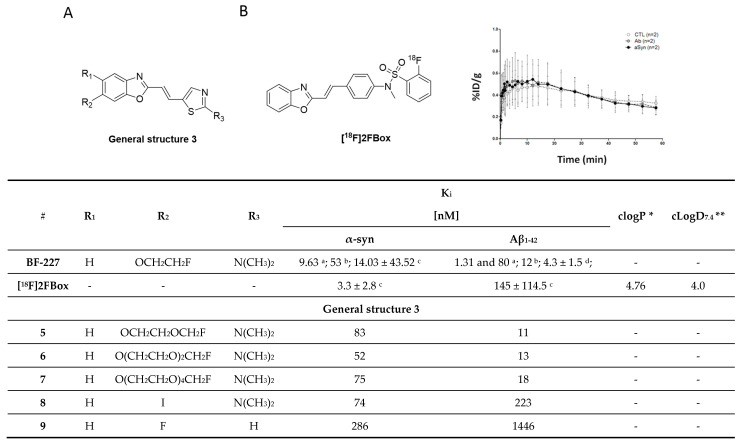
(**A**) General structure of BF-227-like compounds with binding properties specified in table. Affinities were determined for α-syn and Aβ_1–42_ fibrils. Binding affinities were determined by several techniques: ^a^ K_d_ values identified with Scatchard analysis [99,103]. ^b^ In vitro saturation assay using synthetic α-syn and Aβ_1–42_ fibrils [67]. ^c^ In vitro saturation binding assay [67]. ^d^ In vitro binding assays (K_i_) determined for Aβ_1–42_ fibril in competitive binding assay using **[^125^I]BF-180** [98]. * clogP values were calculated by BioByte. ** clogD_7.4_ values calculated by ACD/Percepta. (**B**) The chemical structure of **[^18^F]2FBox** with the TAC from small-animal PET imaging of fibril-injected rats (controls were taken from the striata regions) (reprinted with permission from [67]. Copyright 2018 American Chemical Society).

A series of BF-277-like compounds were designed in order to increase the affinity and selectivity profile of **BF-227** (Figure 10A) toward α-syn. Various oxyethylene groups (**5**–**7**) as well as hydrogen, iodine (**8**) and fluorine (**9**) were introduced to study the substitution pattern of the 6-position of the benzo[*d*]oxazole. Introduction of these oxyethylene groups did not alter the affinity or the selectivity profile of the compound at all. Substitution of the fluoroethoxy group with an iodine increased the selectivity approximately 10-fold, whereas substitution with fluorine or hydrogen lowered the affinity toward α-syn fibrils from 53 nM to 286 nM (Figure 10) [103]. Interestingly, this research suggested that the binding affinity of **BF-227** toward α-syn fibrils was significantly different when compared to the affinity initially reported by Fodero-Tavoletti et al. [99] (Figure 10). A possible explanation for this discrepancy may be that different in vitro assays as well as different fibril preparations were used in these studies.

#### 3.2.3. Rational Drug Design around BF-227

In 2018, Verdurand et al. applied a rational drug design approach to two other lead structure scaffolds (benzoimidazoles and TCAs) based on benzoxazoles. Computational modeling was applied, and various combinations of lead structure motifs were docked into an α-syn fibril model [67]. Among the 10 virtually designed compounds, two ligands (**2FBox** and **4FBox**) were considered worthwhile to be synthesized and in vitro tested. ^18^F-Radiolabeling of these molecules succeeded with a RCY of 10–19% and an A_m_ = 68–543 GBq/µmol. Affinity and selectivity were determined with an in vitro saturation assay using synthetic α-syn and Aβ_1–42_ fibrils. **[^18^F]2FBox** (Figure 10B) showed the best binding characteristics with an affinity of K_d_ = 3.3 ± 2.8 nM against α-syn fibrils and a roughly 50-fold selectivity over Aβ_1–42_ fibrils. In vitro ARG of rat brains showed that **[^18^F]2FBox** was able to detect α-syn fibrils previously injected. Interestingly, preclinical imaging using the same animal model showed that **[^18^F]2FBox** was not able to image these α-syn fibrils in vivo, although **[^18^F]2FBox** showed sufficient brain uptake and an acceptable, but slow clearance profile. Peak brain uptake was 0.47% ID/g at 12 min p.i. (SUV = 1.6), and the tracer was washed out slowly, lowering its peak uptake only 20% within 50 min. The authors suggested that the discrepancy between the in vitro to in vivo results might be due to the low spatial resolution of PET imaging in comparison to in vitro ARG. The high non-displaceable binding component of **[^18^F]2FBox** could also possibly prevent selective imaging. However, **[^18^F]2FBox** also failed to detect selective binding on postmortem brain tissue (medulla oblongata) from PD and MSA patients using ARG [67].

The discrepancy between results obtained from computational modeling, postmortem human tissue, and recombinant human fibrils are concerning, but support the hypothesis that fibrils formed in vitro (and virtual models thereof), may not sufficiently represent in vivo forms in all cases. Further validation of this hypothesis is urgently needed. Since **2FBox** did not show selective binding to α-syn in postmortem tissue, we believe that **2FBox** is not a promising starting point for the development of suitable α-syn PET radioligands.

### 3.3. Indolinones and Indolinone-Dienes

**Figure 11:**
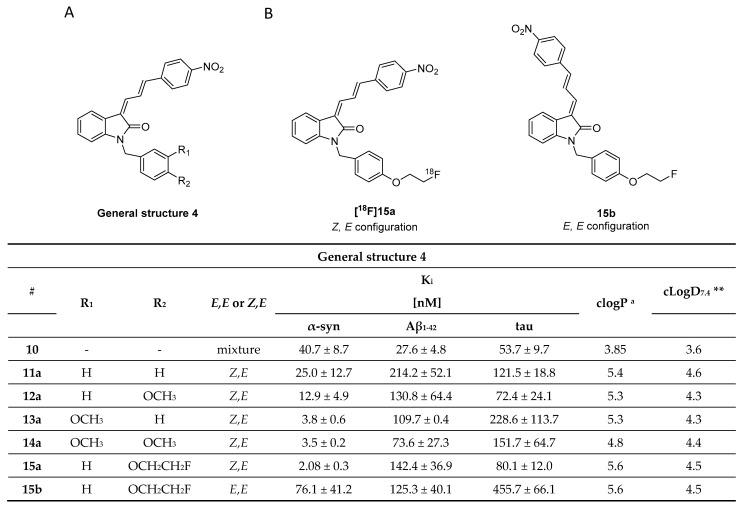
Chemical structures of indolidone-diene analogues with key properties. (**A**) General structure of indolidone-dienes and their binding affinity toward recombinant α-syn, tau, and synthetic Aβ_1–42_ fibrils determined by ThT competitive binding assays [105]. ^a^ clogP were calculated from BioByte software. ** clogD_7.4_ values calculated by ACD/Percepta. (**B**) Chemical structures of **[^18^F]15a** (*Z,E* configuration) and **15b** (*E,E* configuration) [105].

In 2007, Honson et al. reported that indolinone structures confer moderate affinity, but poor selectivity toward α-syn over Aβ_1–42_ and tau fibrils [104]. Neal et al. later demonstrated that the two fluorescent dyes **LDS 798** and **LDS 730** (Figure 5A) were capable of detecting LB in postmortem PD tissue [87]. This inspired Chu et al. to combine the key features, namely the double bond fragment of the dyes and the indolinone structure, into one new lead. With these modifications, they hoped to increase the selectivity toward α-syn [105]. A series of over 40 compounds was synthesized, and the novel indolinone-diene derivatives displayed higher selectivity toward α-syn. Single double bonds only showed moderate affinity and no selectivity while diene analogues showed improved affinity and selectivity. We speculate that the increased length of these diene analogues is responsible for this and allow for a stronger interaction between the ligand and the binding site. The *Z, E* configuration was shown to be more active (Figure 11A,B). Five indolinone-diene analogues showed an affinity of K_i_ < 25 nM toward α-syn fibrils and > 5-fold selectivity over Aβ_1–42_ and tau fibrils. Compound **15a** appeared to be most promising with the highest observed affinity for α-syn (K_i_ of 2.08 nM) and an approximately 68-fold and 40-fold lower affinity for Aβ and tau fibrils, respectively. Affinities were measured using a ThT based competitive binding assay. These promising data prompted Chu et al. to ^18^F-radiolabel **15a** (Figure 11B) resulting in an isolated activity of 0.71 ± 0.23 GBq (decay corrected) and an A_m_ ranging from 29.6 to 185 GBq/µmol. K_d_ measurements of **[^18^F]15a** confirmed the affinity and selectivity profile measured by aforementioned competition binding assay, i.e., **15a** can selectively be displaced from its binding site [43]. Binding affinities toward α-syn, Aβ and tau fibrils were determined to be 8.9, 271 and 50 nM, respectively and **15a** was identified to preferential bind to BS 3/13 of α-syn fibrils [43].

**Figure 12:**
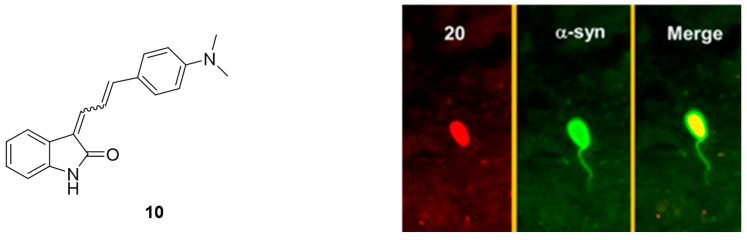
Chemical structure of **10** and fluorescent microscopy studies on brain tissue from PD/DLB and PD patients (reprinted with permission from [105] Copyright 2015 American Chemical Society).

Despite these encouraging results, **[^18^F]15a** has not been tested on patient-derived tissues, and the compound has not been thoroughly evaluated in preclinical PD animal models. The authors reasoned that **15a** possesses a logP of 4.18, which is outside of the standard range for CNS tracers [80] and furthermore, they were concerned that the nitro-group of **15a** could be reduced in vivo [105]. A limited in vivo evaluation in NHP was nevertheless conducted and [**^18^F]15a** showed slow clearance [54]. Since **15a** is one of the most selective α-syn ligands ever designed, we believe this compound should have been tested in preclinical PD animal models and in vitro on patient-derived tissues. Why another compound with lower affinity and selectivity, namely compound **10**, was instead evaluated on brain tissue from PD/DLB and PD patients with fluorescence microscopy (Figure 12) remains unclear. Unfortunately, *E,E* and *E,Z* stereoisomers were not isolated, and the compound was applied as a mixture with a 1:1 ratio. **10** was clearly able to stain LB. Aβ plaques from AD patients may also be stained, bringing the α-syn selectivity of **10** into question [105]. Nevertheless, this study showed that radiolabeled derivatives from this structural class are able to detect α-syn fibrils only when the latter are present in high concentrations. Future studies based on **15a** are warranted and should focus first on the in vitro binding profile of patient-derived α-syn tissue at low concentration. If promising, extensive SAR studies should be conducted to develop better compounds with improved pharmacodynamics and α-syn selectivity.

### 3.4. Thiazole Derivatives

The thiazole moiety is a widely applied scaffold to enhance affinity and selectivity of potential α-syn targeting compounds. For instance, the benzothiazole ring is present in cyanine dyes (**T-284** and **SH-516**, Figure 5) and fluorescent dyes (**ThT**, Figure 5) that have previously been shown to bind to α-syn fibrils. In previous sections, the thiazole moiety already appeared for example within the ligand **BF-227**. In this section, we will further discuss its past role in the design of a selective α-syn ligand.

#### 3.4.1. Diphenylthiazole Derivatives

**Figure 13:**
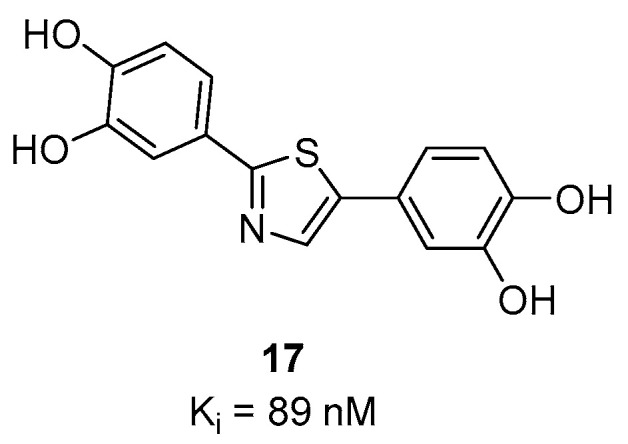
Chemical structure of **17** with binding affinity K_i_ = 89 nM [106].

Several diphenylthiazole, diphenyloxazoles and diphenylthiophenes compounds were developed as potential α-syn imaging agents [106]. However, only one diphenylthiazole, compound **17** (Figure 13) has thus far shown a reasonable affinity (K_i_ = 89 nM) toward α-syn aggregates in affinity measurements conducted using a competition binding assay. Compound **17** also did not show any significant binding for Aβ in AD tissue.

#### 3.4.2. Bithiazole Derivatives

**Figure 14:**
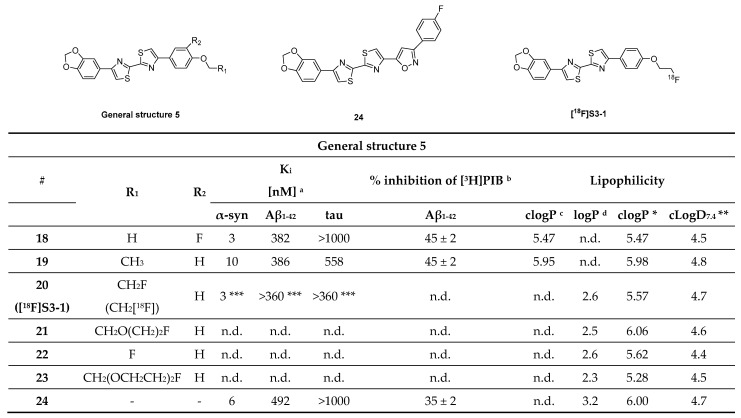
Chemical structures of bithiazole derivatives with key properties. Chemical structure of potential α-syn ligands with binding properties toward recombinant α-syn, Aβ_1–42_, and tau fibrils. ^a^ Affinity toward α-syn fibrils, synthetic amyloid peptides (Aβ_1–42_) and human recombinant tau-441 determined by saturation binding assay [107]. ^b^ % inhibition of **[^3^H]PIB** determined by competition binding assay [107]. ^c^ calculated clogP according to the reference [107]. ^d^ Measured logP from octanol/PBS study according to the reference [107]. n.d.: not determined. * clogP values were calculated by BioByte. ** clogD_7.4_ values calculated by ACD/Percepta. *** Literature source [108].

Bithiazole derivatives were reported as promising new ligands for the detection of neuropathological aggregates, including α-syn. Unfortunately, the only available literature source, to date, is patented and no additional articles are available on the subject. Researchers from the Technical University of Munich developed these bithiazoles and among them compounds **18**, **19** and **24** (Figure 14) showed high affinity for α-syn and good selectivity over Aβ_1–42_ and tau fibrils (as determined by saturation binding assays). Several bithiazoles from the same family were identified as potential ligands for targeting Aβ and tau. Potential ligands for α-syn are summarized in Figure 14 with limited binding affinity data. Compound **18** demonstrated the highest affinity (K_i_ = 3 nM) and a selectivity of 127-fold and >300-fold over Aβ_1–42_ and tau, respectively. Additionally, compounds **20**, **21**, **22** and **23** (Figure 14) were also reported to be selective for α-syn, although no binding data was published [107]. Just recently, **[^18^F]S3-1** (Figure 14) a novel structurally related α-syn PET tracer was evaluated in rats, NHPs and humans following a conference abstract [108]. **[^18^F]S3-1** showed binding affinity of 3 nM for α-syn and high selectivity of 120-fold for Aβ and tau. Brain kinetics were investigated in E46K rats demonstrating statistically significant difference between E46K and health controls. Furthermore, in NHPs initial brain update was up to 1.4% ID and the SUV was 1.3 at 30 min with rapid clearance (SUV ~ 0.1) but with marginally increases in the later timepoints (SUV = 0.4 at 120 min). Dynamic PET studies showed specific binding of **[^18^F]S3-1** in patients with underlying α-synucleinopathies [108]. In summary, bithiazole derivatives present a class of compounds with excellent affinity and selectivity, however more detailed scientific literature is needed in order to provide more thorough conclusions.

#### 3.4.3. Benzothiazole Derivatives

**Figure 15:**
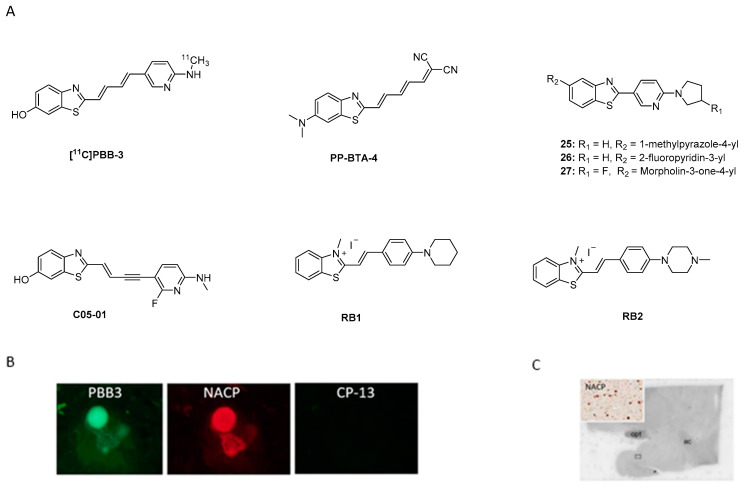
Chemical structures and examples of fluorescence and ARG. (**A**) Chemical structures of **[^11^C]PBB-3**, **PP-BTA-4**, compounds **25, 26, 27**, and **RB1**, **RB2**. (**B**) PBB-3 fluorescence labeling and immunofluorescence double staining for α-syn (NACP) and phosphor-tau (CP13) in DLB from brainstem type LB (reprinted with permission from [111]. Copyright 2017 International Parkinson and Movement Disorder Society). (**C**) Autoradiographic labeling using **[^11^C]PBB-3** in amygdala of DLB patients (reprinted with permission from [111]. Copyright 2017 International Parkinson and Movement Disorder Society).

Benzothiazole analogues have recently attracted attention as potentially selective α-syn PET ligands. Among this class, **[^11^C]PBB-3** was initially developed to image namely tau aggregates [109]. Its binding affinity toward tau was 1.8 nM (neocortex/hippocampus transgenic mice) [110]. During preclinical evaluation, binding toward α-syn was detected. Koga et al. were the first to examine the ability of **[^11^C]PBB-3** (Figure 15A) to bind to brain-derived LB, LN, and glial cytoplasmic inclusions (GCI) from patients with α-synucleinopathies (MSA and DLB). Fluorescence imaging showed that **PBB3** can bind to α-synucleinopathies only at high concentrations (32.3 µM). For example, immunofluorescence double-staining clearly demonstrated this binding (Figure 15B). In ARG and at a low concentration (10 nM, A_m_ = 133 GBq/µmol), **[^11^C]PBB-3** could only bind to the high density GCIs from MSA patients. No specific binding was detected in DLB patients (Figure 15C) [111]. Recently, an additional compound based on the benzothiazole scaffold was identified. **PP-BTA-4**, a push-pull (PP) benzothiazole derivative, was shown to bind to α-syn. **PP-BTA-4** (Figure 15A) strongly increased its fluorescence intensity upon binding to the protein aggregates in solution. A K_d_ of 48.0 ± 0.6 nM was determined by an in vitro saturation binding assay on α-syn aggregates from human PD brains. **PP-BTA-4** also bond to Aβ_1–42_ (derived from AD patients) with an affinity in the same order of magnitude [112]. Other benzothiazole derivatives of interest are compounds **25**, **26**, and **27** (Figure 15A). In a fluorescence-based assay using amygdala brain sections derived from fully developed PD and AD patients (Braak stage V -VI) selective binding of these compounds to α-syn over Aβ plaques was detected. The compounds were incubated on the brain sections at a high concentration of 100 µM. Further binding affinity measurements revealed K_d_ values for compounds **25** and **26** of 50 nM and 14 nM, respectively [113]. Affinities were determined by backscattering interferometry on human PD brain homogenates (cortex). Affinity data for **27** is not reported [113]. Just recently, a novel analogue of PBB3, **C05-01** (Figure 15A), has been discovered showing a K_i_ of 3.5 nM for brain homogenates and a K_i_ of 25 nM for α-syn fibrils. **C05-01** was derived from a library of 44 compounds that were structurally similar to PBB3 and was selected based on quantitative evaluation using fluorescence microscopy and a semiquantitative approach using a tissue microarray (TMA).

TMA is an effective approach for high-throughput molecular analysis of pathological tissues, as it makes use of paraffin blocks, which allow one to measure several tissue cores without consuming the tissue block. A further advantage of TMA is that it can be used to examine tissue from multiple patients. TMA also allows multiplexing of histological analysis including immunohistochemistry, immunofluorescence, histological staining. The binding profile of **C05-01** was determined on recombinant α-syn fibrils using saturation and competition binding assay with **[^3^H]C05-01** and **C05-01** and an affinity in the range 25–30 nM was determined. Further studies using brain homogenates from DLB (amygdala) revealed different affinities to those previously reported by Koga et al. [111]. **[^11^C]PBB3** showed a moderate affinity (K_d_ of 58 nM) while non-labeled **C05-01** demonstrated a high affinity (K_i_ of 3.5 nM). These data once again showed that the tertiary structure of fibrils and brain homogenates can be different. Another reason for the observed discrepancy may be the variance of experiments. Further characterization of selectivity using PD, MSA, AD and Pick’s disease TMA sections and fresh frozen tissue confirmed the binding of **C05-01** to α-syn, however, binding to other amyloids was also shown [114]. Recently, new ThT analogues, namely **RB1** and **RB2** (Figure 15A), were developed in order to achieve stronger selectivity for α-syn over amyloid fibrils. Key structural features include introduction of a double bond and cyclic nitrogen donors (either piperidine or piperazine). Binding affinities were tested using α-syn fibrils (concentration < 30 µM) and revealed K_d_ values of 30 nM and ~4.4 µM for **RB1** and **RB2**, respectively. The authors hypothesize that the piperidine moiety fits better in the hydrophobic pocket of α-syn fibrils and therefore **RB1** shows higher affinity for α-syn than the lead compound. No selectivity data over Aβ or tau have been reported. Encouraged by these results the authors studied **RB1** and its applicability for live-cell imaging using the SH-SY5Y human neuroblastoma cell line. Cultured cells were incubated with short α-syn fibrils. Then, these cells were incubated with **RB1** at concentration 500 nM and demonstrated selective staining of α-syn fibrils in the cytosol. This study showed an approach to implement the design and development of new compounds. However, binding affinity and selectivity profile need to be further improved and investigated [115].

In conclusion, benzothiazole derivatives appear to be structural leads which may be help in the development of an α-syn selective compound. Extensive SAR studies are still needed to learn more about how binding and selectivity can be improved.

#### 3.4.4. Thienopyridine, Thiazolo Pyridine Derivatives

**Figure 16:**
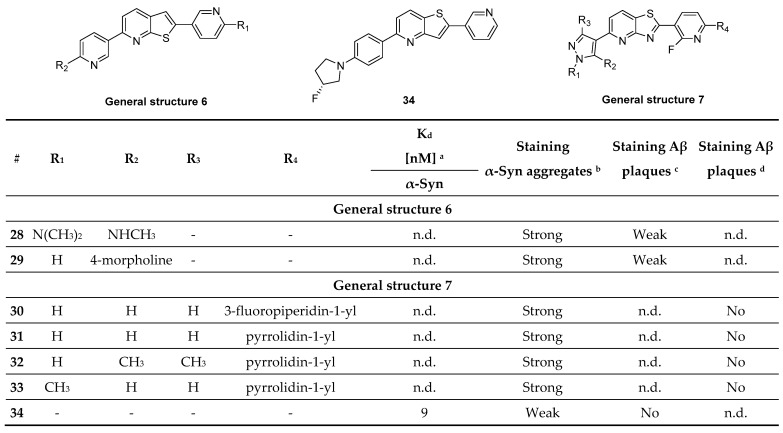
Chemical structures of thienopyridine, thiazolo pyridine derivatives with key properties. Chemical structures of compounds **28**–**34** and general structures with key binding properties. ^a^ Affinity toward α-syn fibrils aggregates determined by backscattering interferometry on PD brain homogenates. ^b^ Staining of α-syn aggregates on PD sections. ^c^ Staining of Aβ plaques on AD sections. ^d^ Staining of Aβ plaques on PD sections with mixed pathology. n.d.: not determined. Literature source [113].

Recently, a new heterogeneous class of compounds was discovered, which may stain α-syn aggregates as well as Aβ plaques. Fibrils were derived from fully developed PD and AD patients (Braak stage V-VI) and a library of 130 compounds were incubated at 100 µM and their neuropathological staining was confirmed by staining with primary antibodies. Of the 130 tested compounds, only several (**28**–**34**) showed selectivity for α-syn aggregates over Aβ plaques (Figure 16, determine with fluorescence staining). The only compound that was tested for affinity was compound **34**, although it only shows weak staining of α-syn aggregates. This decision was most likely taken since **34** did not stain Aβ plaques. An affinity (K_d_) of 9 nM was determined by backscattering interferometry on human derived tissue (PD brain homogenates) [113]. To further evaluate this class of compounds, a comprehensive in vivo evaluation, in vitro ARG at lower concentrations and radiolabeling studies should be performed.

### 3.5. Benzoimidazole Derivatives

#### 3.5.1. Styryl Benzodiazole Derivatives

**Figure 17:**
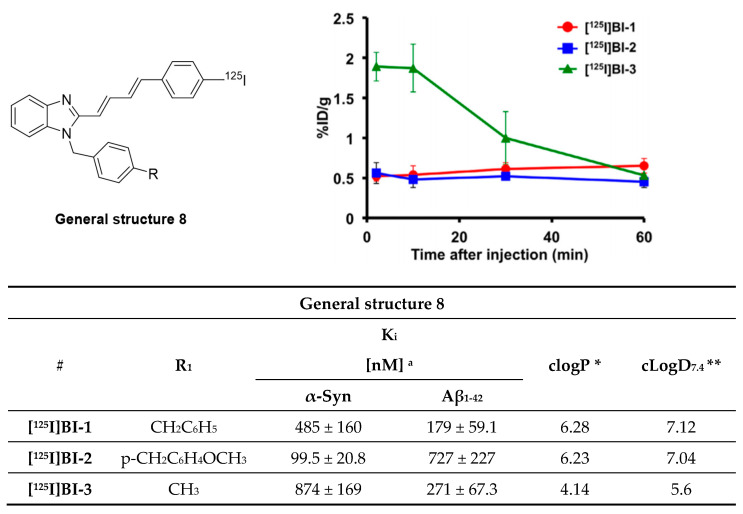
General structure of radio-iodinated benzoimidazole derivatives and their brain uptake after intravenous injection. Binding affinities were determined on recombinant α-syn and Aβ_1–42_ aggregates. (Reprinted with permission from [117]. Copyright 2017 Elsevier). * clogP values were calculated by BioByte. ** clogD_7.4_ values calculated by ACD/Percepta.

Styryl benzazole derivatives have been shown to interact with different amyloid fibrils [116]. This inspired Watanabe et al. to test if this structural class of compounds can be used to develop an α-syn selective compound [117,118]. Three novel probes were synthesized and subsequently labeled with iodine-125 (Figure 17). Watanabe et al. speculated that incorporation of dienes in the linker would enhance the affinity toward α-syn aggregates and that bulky substituents like benzyl groups might increase selectivity toward α-syn over Aβ aggregates. These considerations followed the theory developed by Chu et al. during the development of compound **15a** [105]. Saturation binding assays based on fluorescence intensity measurements on recombinant α-syn and Aβ_1–42_ aggregates showed that the developed probes possessed a Kd in the range of 99.5–874 nM. **[^125^I]BI-2** showed the best affinity and selectivity profile (K_d_ 99.5 ± 20.8 nM and 7-fold selective over Aβ_1–42_). These results suggest that bulky substituents enhance affinity as well as selectivity. Consequently, **BI-2** was studied further using fluorescent staining on PD and AD brain sections (at 200 µM). Stained sections were compared with immunohistochemistry and co-localization was observed in the case of α-syn positive tissue, but not for Aβ positive tissue. These results encouraged the research to label two additional derivatives of **BI-2** and evaluate the in vivo biological behavior of all three compounds. Only **[^125^I]BI-3** showed a moderate brain uptake of 2.0% ID/g at 10 min post injection and moderate clearance within 60 min resulting in 0.5% ID/g (Figure 17) [117]. Further improvements regarding affinity, selectivity and lipophilicity are needed to develop an α-syn selective ligand based on this scaffold.

#### 3.5.2. Azaindole Derivatives

**Figure 18:**
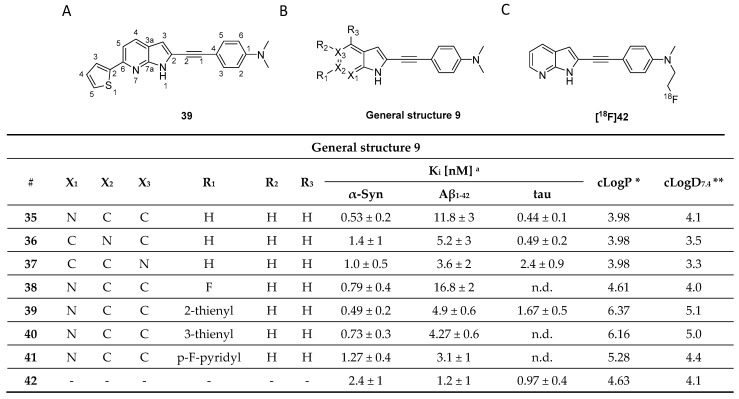
Chemical structures of azaindole derivatives with key properties. (**A**) Chemical structure of compound **39**. (**B**) General structure of azaindole derivatives with identified in vitro K_i_ and K_d_ binding properties [119]. (**C**) Chemical structure of **[^18^F]42** that was used for in vivo study. * clogP values were calculated by BioByte. ** clogD_7.4_ values calculated by ACD/Percepta.

A recently published study by Routier et al. reported on a new set of compounds featuring an azaindole moiety and a phenyl ring linked via a triple bond. The process leading to the discovery of these structures was not discussed. Their binding affinity and selectivity were measured by ThT fluorescence assays using synthetic α-syn, Aβ_1–42_, and tau fibrils. Of the nine tested compounds, compound **39** was the most promising ligand (Figure 18A), with a > 22-fold selectivity over Aβ_1–42_ fibrils but only limited selectivity over tau fibrils. SAR studies conducted on the heteroaromatic substituents at the 5-, 6- and 7-position (**35** vs. **36** vs. **37**, Figure 18) of the indole scaffold revealed that the 7-azaindole structure (Figure 18A) resulted in the best selectivity profile. The influence of 2-thienyl, 3-thienyl, p-F-pyridyl, fluoride substitution on the 6-position were also studied (**38** vs. **39** vs. **40** vs. **41**, Figure 18). In particular, substitution with 2-thienyl gave the best affinity but also resulted in limited selectivity. The only compound radiolabeled and evaluated in vivo was **[^18^F]42** (Figure 18C). The in vivo study confirmed it to can cross the BBB despite its relatively high clogD [119]. Notwithstanding the limited amount of peer reviewed literature on this class of compounds, its structural similarities with **C05-01** make it a lead compound for α-syn PET tracer development [114]. Although it is challenging to draw solid conclusions from two different classes of compounds and without any knowledge on their binding profile, we are speculating that the triple bond linker and its induced planarity can enhance the binding of ligands to β-sheets. A similar hypothesis was drawn by Kaide and colleagues [120]. However, due to the structural similarity between different amyloids and their frequent colocalization, high planarity compounds will not be able to discriminate between abnormal aggregates; thus, leading to a moderate to low selectivity profile which is the case for azaindole derivatives. Interestingly, this compound class showed moderate selectivity over Aβ but no selectivity over tau. As such, further SAR studies, in vivo evaluation and ex vivo ARG on postmortem tissue are needed to conclusively determine if azaindole derivatives are promising scaffolds for the future development of α-syn selective ligands.

### 3.6. Diphenylpyrazole Derivatives

**Figure 19:**
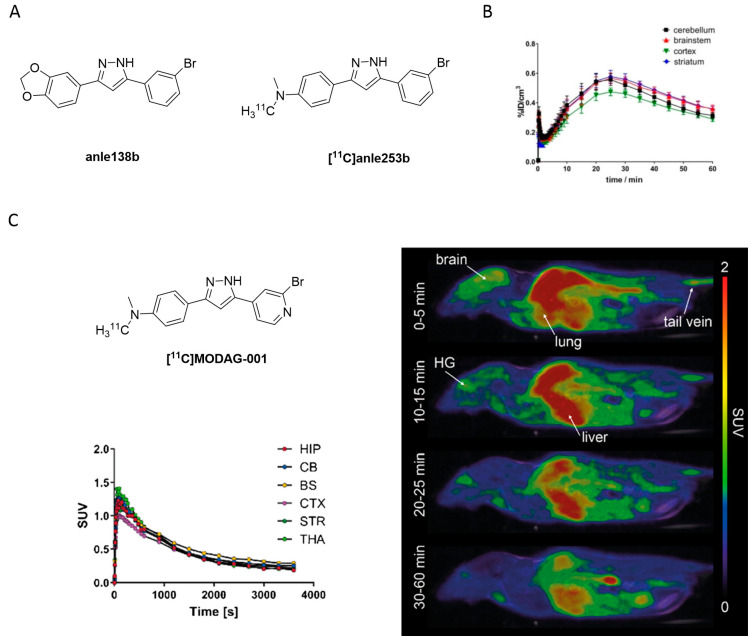
(**A**) Chemical structures of the 3,5-diphenylpyrazole derivatives **anle138b** and **[^11^C]anle253b**. (**B**) TACs of the **[^11^C]anle253b** obtained from the in vivo study in healthy rats (Adapted from [128]). (**C**) Chemical structure of **[^11^C]MODAG-001** with TACs and PET images in mouse (Adapted from [129]).

High-throughput screening of more than 20,000 compounds identified 3,5-diphenylpyrazole (DPP) as a promising small molecule scaffold to detect aggregated α-syn inclusions. DDP based compounds inhibited pathological aggregation of α-syn in vivo by binding to α-syn fibrils as demonstrated by fluorescence spectroscopy. The most promising drug candidate, **anle138b** (Figure 19A) was identified to be an oligomer modulator [121]. Interestingly, Wagner et al. demonstrated that **anle138b** binds only to pathological aggregates of prion protein and α-syn in vivo and not to natural monomers of α-syn [121]. In addition, DPP derivatives were shown to directly bind to α-syn fibrils in vitro [122]. Notably, **anle138b** may inhibit 77% of α-syn oligomer formation, which can be shown in several PD animal models. It also slowed down disease progression. **Anle138b** can be administered orally, crosses the BBB and does not show any significant toxicity after long-term therapeutic application. The intrinsic fluorescence of **anle138b** was used to study its binding properties against α-syn [122]. The fluorescence intensity of **anle138b** increased more than 30-fold after the addition of aggregated α-syn, but did not change after the addition of monomeric α-syn. This change suggested a strong and specific structure-dependent binding of **anle138b**. A K_d_ of 190 ± 120 nM to aggregated α-syn was determined [122]. However, **anle138b** also inhibited the aggregation of other common protein aggregates involved in neurodegenerative diseases [121,123,124,125,126,127]. This selectivity challenge prompted the group to conduct a comprehensive study to increase affinity and selectivity. This led to the identification of **anle253b** [121]. In a competition assay with **[^3^H]anle138b** using recombinant human α-syn fibrils, **anle253b** (Figure 19A) showed a high affinity for α-syn aggregates with an IC_50_ value of 1.6 nM. It also showed preferential binding to α-syn fibrils compared to other α-syn states such as monomers or oligomers. These promising results motivated the carbon-11 labeling of the structure [128]. **[^11^C]anle253b** was synthesized with a high RCY (47%), but with a low A_m_ of 15.1 ± 3.4 GBq/μmol at EOS. Further improvement of the molar activity is most likely needed to optimally image low-density targets such as α-syn aggregates. **[^11^C]anle253b** was evaluated in healthy rats and showed complete excretion after 75 min from the whole animal and low brain uptake with atypical brain kinetics (Figure 19B). For the latter, the authors hypothesized that the high logP (clogP = 5.61 calculated by BioByte) could be the reason. No radiometabolites were detected using radio-HPLC [128]. Presently, no DPP derivatives have been evaluated on human pathological tissues for more accurate understanding of their affinity and selectivity. Recently, a new derivative within this compound class has been reported which has a lower lipophilicity, called **MODAG-001** (clogP = 3.85). **MODAG-001** displayed strong binding to α-syn fibrils (K_d_ = 0.6 ± 0.1 nM), and a moderate affinity to tau (K_d_ = 19 ± 6.4 nM) and Aβ fibrils (K_d_ = 20 ± 10 nM). **MODAG-001** could be isolated in 266 ± 113 MBq and with an A_m_ of 98.6 ± 24.7 GBq/μmol. **[^11^C]MODAG-001** (Figure 19C) showed excellent BBB permeability in mice and was able to visualize fibril-inoculations in rat striata using PET imaging. However, **[^11^C]MODAG-001** was not able to detect aggregated α-syn in human brain sections from DLB patients. Future studies should be designed to reveal the reason for this behavior Furthermore, no evidence for binding toward tau in AD and PSP brain tissues have been detected by ARG. Authors also questioned binding toward Aβ studied in AD brain tissue, however, additional studies should be conducted [129]. Overall, this compound class is considered promising and may represent a lead compound for future SAR studies.

#### Furan-2-yl-1H-pyrazoles

**Figure 20:**
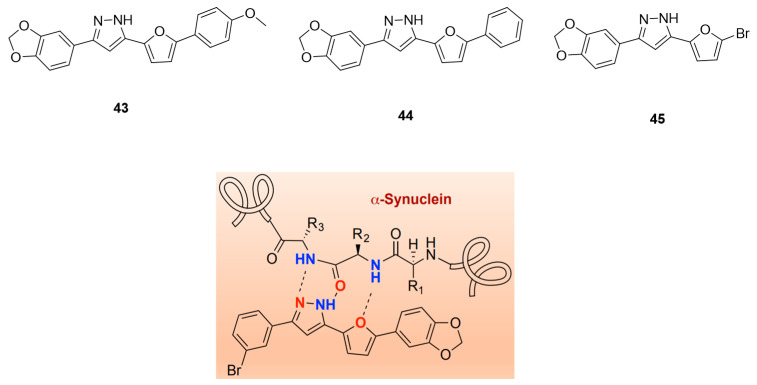
Chemical structures of furan-2-yl-1H-pyrazoles derivatives **43**, **44**, and **45** and proposed binding scheme of a peptide backbone with a furan-2-yl-1H-pyrazole with highlighted HBD and HBA features (reprinted with permission from [130]. Copyright 2020 American Chemical Society).

Inspired by **anle138b** and in particular by the pyrazole ring interactions with the peptide backbone of α-syn (Figure 20), Ryan et al. proposed a novel class of compounds, namely furan-2-yl-1H-pyrazoles. ThT florescence and mass spectrometry (MS) binding assays were performed in order to characterize the inhibitory activity and to understand the binding interactions of this compound class with the protein. As a result, several new compounds were identified. Compound **43** (Figure 20) was identified as a lead pyrazole and compounds **44** and **45** (Figure 20) as lead pyrazolines for further development. These compounds showed the highest ability to inhibit α-syn aggregation. Further in vivo studies are needed to fully characterize them.

### 3.7. Chalcone Derivatives

#### 3.7.1. Radioiodinated Diphenyl Derivatives

**Figure 21:**
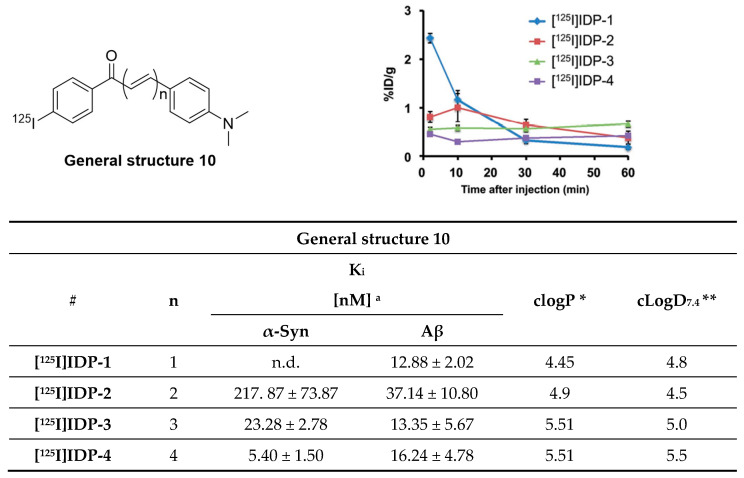
Chemical structure of radioiodinated diphenyl derivatives and brain uptake of radioactivity after intravenous injection. Binding affinity values are determined for recombinant α-syn and Aβ fibrils. Reprinted with permission from Ono et al. [132]. Copyright 2016 Royal Society of Chemistry. * clogP values were calculated by BioByte. ** clogD_7.4_ values calculated by ACD/Percepta.

Chalcone derivatives were originally developed to inhibit the formation of Aβ aggregates [131]. Four chalcone derivatives (Figure 21) were radiolabeled with iodine-125 (A_m_ = 81.4 GBq/µmol, RCY range = 19–60%) and subsequently evaluated for their selectivity profile toward α-syn fibrils [132]. Affinities on recombinant α-syn fibrils in the range of 5.4–217 nM were reported, as well as moderate selectivity over recombinant Aβ fibrils (0.2–3.0-fold). **[^125^I]IDP-4** showed the most promising affinity and selectivity profile with a K_d_ of 5.4 ± 1.5 nM for α-syn and 3-fold selectivity over Aβ. Fluorescent and immunohistochemical staining on human brain slices showed that **IDP-4** (at 20 µM) was able to visualize α-syn and LBs. **IDP-3 and IDP-4** displayed a similar binding pattern. In contrast, **IDP-1** and **IDP-2** did not show specific α-syn binding on post-mortem brain samples from PD patients. All compounds were evaluated in vivo. **[^125^I]IDP-1** had an initially high brain uptake (2.43% ID/g), while all other derivatives displayed low brain uptake (range 0.45–0.81% ID/g) at 2 min p.i. as exemplified in Figure 21. The reason for the relatively low brain uptake of **[^125^I]IDP-4** is unknown but may be attributed to its high molecular weight [132]. Further improvement with respect to selectivity and affinity are most likely needed to selectively image α-syn depositions.

#### 3.7.2. Chalcone and Five-Membered Heterocyclic Isosteres

**Figure 22:**
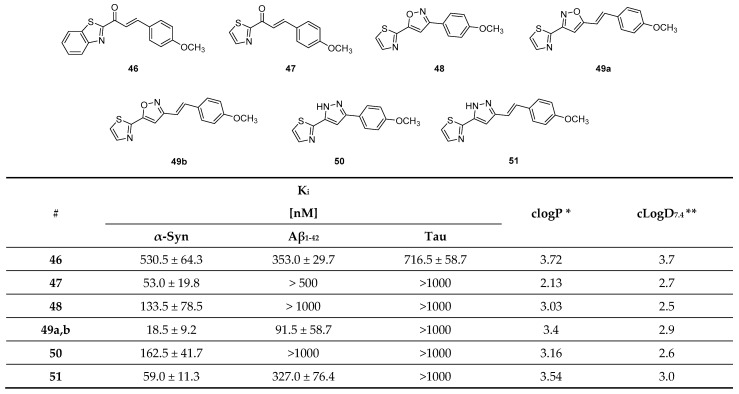
Chemical structures of chalcone derivatives and their affinity values expressed as K_i_ values toward α-syn, Aβ and tau fibrils [133]. * clogP values were calculated by BioByte. ** clogD_7.4_ values calculated by ACD/Percepta.

Hsieh et al. attempted to diversify the structure of the chalcone core by substituting the phenyl group of the “benzoyl moiety” with a 2-benzothiazolyl and a 2-thiazolyl group (compounds **46**, **47**, Figure 22) in the development of an α-syn PET tracer. Surprisingly, the five-membered heterocyclic isosteres conferred much higher α-syn selectivity than the benz-fused aromatic constituent. Substitution of the “styrene moiety” at 4-position had a major influence on the affinity and selectivity profile of the compound [133]. Methoxy substitution resulted in the highest affinity and selectivity for α-syn, and further SAR studies were centered around this key structure. For example, replacement of the enone moiety with an isoxazole (**48**, Figure 22) or a pyrazole structure (**50**, Figure 22) was investigated. Both of these derivatives were further modified via extension with a styrene moiety which was substituted with a methoxy group at 4-position. A ThT competition binding assay performed with these compounds revealed that the isoxazole and pyrazole derivatives with a double bond (compounds **49**, **51** in Figure 22) bind with higher affinity to α-syn fibrils compared to their analogues without the double bond linker. They also displayed a higher affinity for Aβ fibrils, whereas the affinity toward tau fibrils were not altered [133]. Subsequently, a molecular-modeling approach was conducted to study which properties influenced the binding of these structure toward α-syn, Aβ or tau fibrils. 3D geometric characteristics such as the shape of the molecule (linearity and flatness), topological diameters or intramolecular distance between hydrogen bond receptors were calculated for various structures. Interestingly, the order of binding affinity (**49b** > **47** > **48)** correlated to the order of distance between the isoxazole oxygen and the methoxy group (9.98 > 8.44 > 7.72 Å). Moreover, it appeared that compounds with higher degrees of flatness and linearity such as isoxazole and pyrazole analogues showed increased affinity for α-syn [133]. Finally, compounds **49b** and **51** displayed a preference for BS2 as indicated by molecular docking [43]. In general, the presented structures display good affinity and selectivity over amyloid fibrils. For example, the two isoxazole derivatives **49a** and **49b** showed a ~50 fold selectivity over tau fibrils and moderate affinity (18.5 ± 9.2 nM) toward α-syn fibrils. Presently, none of these compounds have been radiolabeled and evaluated on human brain tissue or in in vivo animal models. More comprehensive studies on this family of compounds should be conducted.

### 3.8. Quinolinyl Analogues

**Figure 23:**
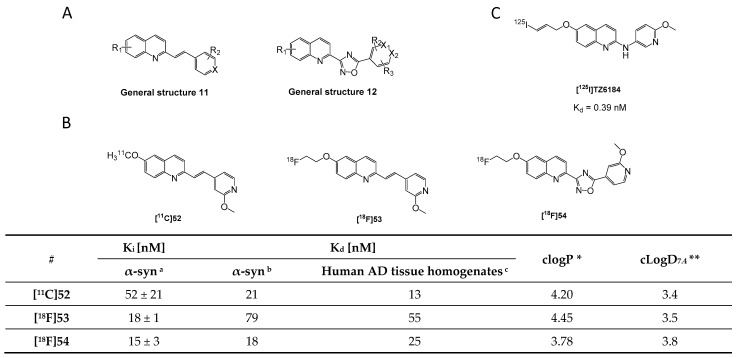
Chemical structures of new quinolinyl analogs. (**A**) General structures 11 and 12 of quinolinyl analogs. (**B**) Chemical structures of new radiolabeled quinolinyl analogs **[^11^C]52**, **[^18^F]53** and **[^18^F]54**. ^a^ Binding affinities expressed as K_i_ values determined by ThT indirect competitive binding using recombinant α-syn fibrils. ^b^ Binding affinities expressed as K_d_ values and determined by direct radioligand competitive binding using recombinant α-syn fibrils. ^c^ Binding affinities expressed as K_d_ values and determined by direct radioligand competitive binding assay on tissue homogenates from AD patients [134]. * clogP values were calculated by BioByte. ** clogD_7.4_ values calculated by ACD/Percepta. (**C**) Chemical structure of [^125^I]TZ6184 with binding affinity K_d_ = 0.39 nM [135].

Several compounds containing a quinolinyl moiety and six-membered aromatic rings linked either by a double bond or an oxadiazole bridge were designed by Yue et al. for α-syn imaging [134]. A rational design approach was applied based on previous findings combining key features of the compounds **LDS 798** and **2,6-ANS** (Figure 5). In particular, the incorporation of double bonds into the side chain, the addition of heteroatoms on both aromatic rings, the use of secondary amines or oxadiazole fragments to bridge both aromatic rings were explored (Figure 23A). As a result, 25 new quinolinyl analogs were synthesized and tested in vitro. Only three derivatives displayed K_i_ values < 52 nM. Affinity measurements were conducted using a ThT competitive binding fluorescence assay with recombinant α-syn fibrils. Despite these moderate affinities, all three compounds (**52**, **53**, **54**) were radiolabeled with carbon-11 or fluorine-18 (Figure 23B). Using recombinant α-syn fibrils and brain AD tissue homogenates, the affinity of these compounds was determined to be 21, 79, 18 nM for **[^11^C]52**, **[^18^F]53**, **[^18^F]54**, respectively. Affinity toward Aβ fibrils was determined to be in the same order of magnitude [134]. These binding characteristics discouraged the authors from further evaluating the compounds in vivo. Recently, another quinolinyl analogue **[^125^I]TZ6184** (Figure 23C) has been reported with limited information. In vitro characterization of this radiotracer on recombinant α-syn fibrils showed that it possesses an extremely good K_d_ of 0.39 nM [135]. However, no selectivity profile has been reported and thereby no thorough conclusions can be made.

### 3.9. N-Substituted Phenyl Amides

#### 3.9.1. *N*-Phenylbenzamide Analogues

**Figure 24:**
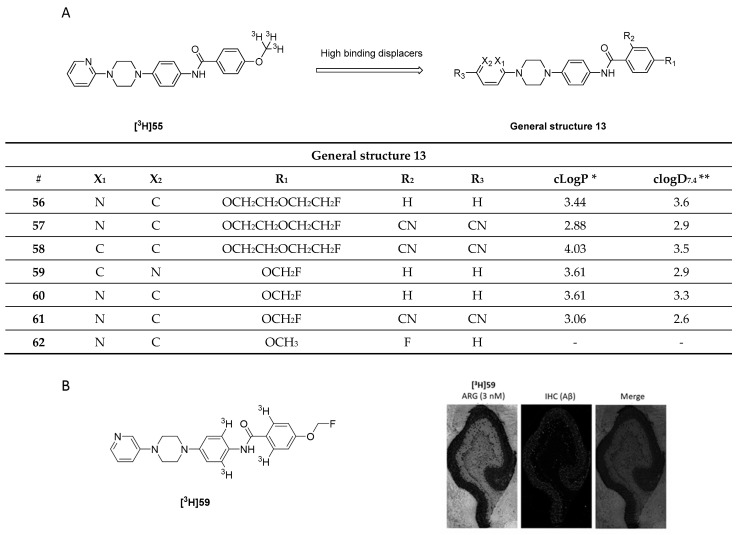
Chemical structures of *N*-phenylbenzamide analogues. (**A**) General structure of *N*-phenylbenzamide analogues (general structure 13) that showed to be high affinity displacers and chemical structure of **[^3^H]55**. Key properties are summarized in table below [136]. (**B**) Chemical structure of **[^3^H]59** and its binding to human AD brain tissue (cortical tissue) and comparison with staining of anti-Aβ mAb (adapted from Borroni et al. [136]). * clogP values were calculated by BioByte. ** clogD_7.4_ values calculated by ACD/Percepta.

In a series of over 170 compounds, a new class of α-syn binders was identified by Borroni and colleagues [136]. The initial idea about the molecular design of these *N*-phenylbenzamide derivatives is not disclosed in the patent where these molecules are presented. The binding profile of **[^3^H]55** (Figure 24A) was studied by ARG on A30P transgenic mouse and human PD brain sections, and the results encouraged the authors to use this compound for displacement studies for a set of other *N*-phenylbenzamides. Results are expressed as % displacement. Six compounds (**56**–**61**) were identified as high affinity ligands with a displacement of >87% (in A30P mice, at 1 µM), which were tritiated to evaluate their binding profile in ex vivo ARG in A30P mice. High brain accumulation as well as good target engagement in the midbrain, pons and subthalamic brain regions was detected. No significant off-target binding was reported. The selectivity of all tritiated compounds was assessed with an in vitro displacement assay using Aβ AD human brain tissue. The experiments showed that all compounds also bind to Aβ (Figure 24B). As such, further structure activity studies are needed to increase the selectivity of these compounds to α-syn [136]. Finally, their binding interactions with α-syn was studied using molecular docking techniques, suggesting that the compounds bind preferentially within BS 9 [43].

#### 3.9.2. *N*-Substituted Phenyl Amides Analogues

**Figure 25:**
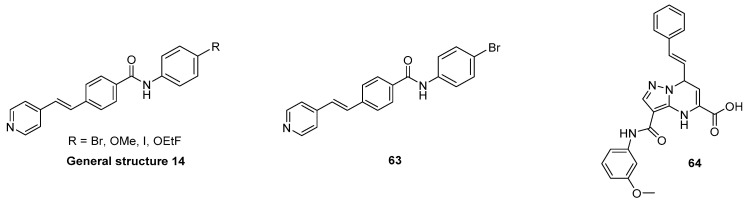
Chemical structures of *N*-substituted phenyl amide analogues. From left to right: general structure 14 of *N*-substituted phenyl amide analogues. Chemical structure of compounds **63** and **64**.

Several *N*-substituted phenyl amide derivatives bearing phenothiazine, phenoxazines, quinolinyl, and styryl pyridinyl substituents were also reported to be potential α-syn binders [94]. Moderate binding affinity with K_i_ values < 100 nM were determined by a ThT competitive assay on α-syn fibrils. *N*-substituted phenyl amides appeared to be most promising (Figure 25) and were studied further. Substitution at the 4-position of the phenyl moiety showed that the binding affinity is reduced in the sequence: Br > OMe > I > OEtF. Compound **63** (Figure 25) showed the highest affinity (ca. 10 nM). No further studies concerning selectivity or in vivo evaluation have been reported up till now [94]. In a different study, Xu et al. used automated MS as a technique to quickly detect the direct interaction between molecules and proteins. Using this method 2500 compounds were screened for α-binding and resulted in one hit: compound **64** (Figure 25) Furthermore, the increase of fluorescence during aggregation process was evaluated during the ThT assay, showing that compound **64** was able to strongly inhibit α-syn aggregation by 91% [137]. Currently, there are no known PET tracers developed from compound **64** and no binding affinity or selectivity profile are known. Thus, additional studies are needed.

#### 3.9.3. 2-Phenoxy-*N*-(3-phenylisoxazol-5-yl)acetamide Analogues 

**Figure 26:**
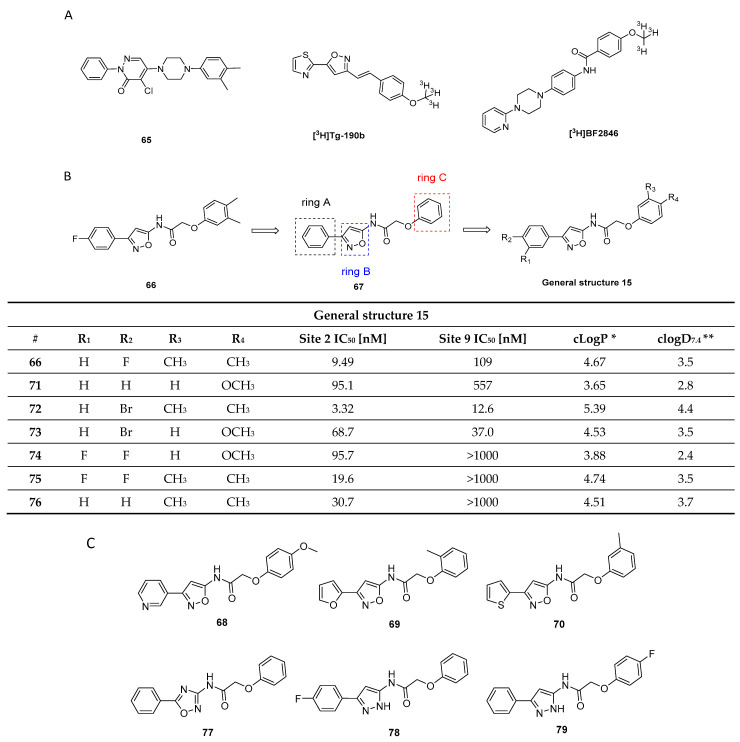

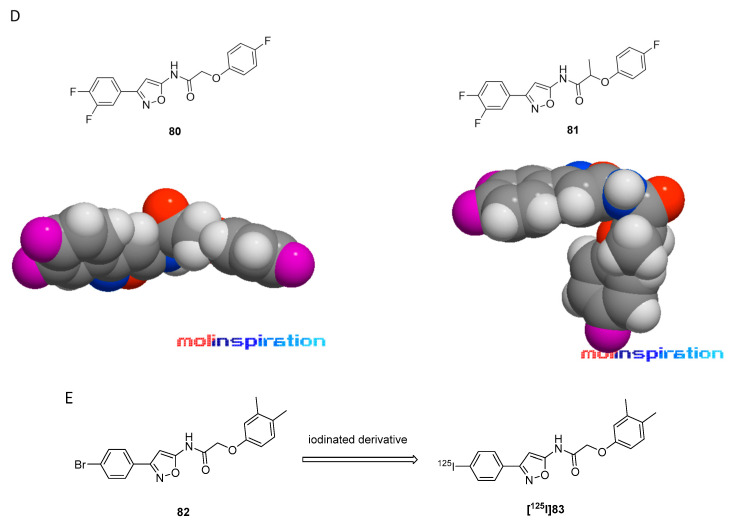
Chemical structures of 2-phenoxy-*N*-(3-phenylisoxazol-5-yl)acetamide analogues. (**A**) Chemical structure of compounds **65**, **[^3^H]Tg-190b** and **[^3^H]BF2846**. (**B**) Chemical structure of compounds **66**, **67** and general structure of most potent α-syn binders. Table represents IC_50_ values screened for BS2 and BS9 using **[^3^H]Tg-190b** and **[^3^H]BF2846** [139]. * clogP values were calculated by BioByte. ** clogD_7.4_ values calculated by ACD/Percepta. (**C**) Chemical structures of compounds **68**–**70**, **77**–**79** (**D**) Models of likely 3D structures of compounds **80** and **81** (visualized by Molinspiration Galaxys 3D Structure Generator v2018.01 beta). Methyl substituent of compound **81** generates non-planar structure in comparison with compound **80**. (**E**) Chemical structure of compound **82** and **[^125^I]83**.

An exemplar-based in silico screening campaign was used to identify potential ligands for α-syn. An exemplar is defined as an ideal pseudoligand that would optimally fit in the protein surface pocket; therefore, it is exploited as a template for pharmacophore screening [138]. An in silico assay was designed to highlight compounds that bind to two previously identified binding sites—BS2 and BS9—within the α-syn fibrils (2n0a, Figure 2C). Approximately 10 million molecules from the ZINC15 database were screened and led to the identification of 17 chemically diverse potential α-syn binders. Compounds **65** and **66** (Figure 26A,B) were of special interest since they were able to displace **[^3^H]Tg-190b**—a selective ligand for binding site BS2 (Figure 26A)—from α-syn fibrils. Inhibitory concentrations, IC_50_ values, were 490 nM and 9.49 nM for **65** and **66**, respectively. Compound **66** was used as a lead and the molecule was deconstructed to identify the essential features needed for binding to α-syn. The new resulting scaffold was compound **67**—a 2-phenoxy-*N*-(3-phenylisoxazol-5-yl)acetamide. This template was then used to find structurally similar compounds from the Mcule compound library. 39 promising structures were discovered using this approach. All compounds were tested using the aforementioned competition binding assay with **[^3^H]Tg-190b** and **[^3^H]BF2846** (Figure 26B). More important SAR were obtained when different heterocycles were introduced to the core structure within rings A, B or C (Figure 26B). For example, replacement of the A ring with a pyridine (**68**), furan (**69**) or thiophene (**70**) group did not improve binding. However, we were not able to draw these conclusions since the identical compounds with phenyl moiety as the A ring, for compounds **69** and **70**, were not presented in the publication. Conversely, *para* substitution on the A ring improved binding toward α-syn fibrils in many cases (see for example **76** vs. **66**). Also, the electronic nature of ring A showed to be essential. In particular, a bromo substituted version (**73**) showed to have a higher affinity than **71**, when a methoxy group is present on ring C. Also, comparing compounds **72** and **66** revealed that a bromo substituent has a significant effect on binding affinity. Moreover, the authors studied the influence of additional fluoro substituents (**75**) which showed to have a positive effect on affinity (**75** vs. **76**).

Replacement of the B ring with an oxadiazole heterocycle lowered the affinity. All compounds possessing an oxadiazole (for example **77**) showed no displacement of **[^3^H]Tg-190b** (Figure 26C), no matter the substituents on rings A or C. In contrast, replacement of B with a pyrazole only increased the affinity moderately (compounds **78** and **79** (Figure 26C). In addition, from the comparison of heterocyclic differentiations in the B ring we cannot conclude which of the heterocyclic rings had the best influence on affinity. Substitution on the C ring revealed the following: *ortho* substituents decrease binding, while *meta* and *para* substituents improve binding. In addition, the electronic nature of the C ring for *para* substituents is essential. Since *para* substituents and in particular fluoro substituents show decreased binding, while moderate to strong binding is demonstrated by derivatives bearing electron donating groups such as methyl and methoxy groups. As a general trend, non-planar compounds showed weaker binding than the planar compounds exemplified in Figure 26D (**80** and **81**) [121,139]. Similar observations were previously drawn for other structural classes. The most promising compound identified in this study was **82** (Figure 26E). It displayed an IC_50_ of 3.32 nM and of 12.6 nM for BS2 and BS9, respectively. The iodinated derivative **83** (Figure 26E) was selected for labeling with iodine-125. Binding experiments with **[^125^I]83** (RCY = 57%, A_m_ = 81 GBq/µmol) on α-syn fibrils demonstrated excellent binding (K_d_ = 1.06 nM), but only moderate selectivity over Aβ_1–42_ fibrils (4-fold, K_d_ = 4.56 nM). Binding to other proteins in mouse brain lysate was found to be high. In vitro ARG was performed using brain tissue from A53T PD mice (15 months old). Images confirmed binding of **[^125^I]83** to α-syn rich brain regions [139]. Further efforts are needed to decrease unspecific protein binding and increase selectivity. Additional data for human tissue are highly needed to further develop this class of molecules.

### 3.10. Flavonoids

**Figure 27:**
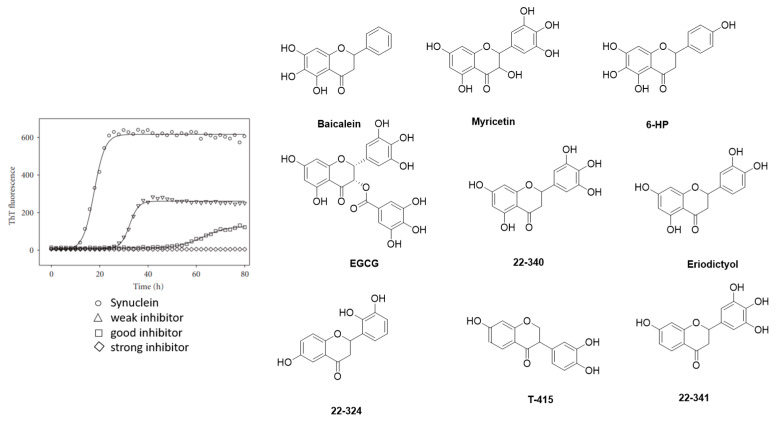
Kinetic profiles of the fibril formation (adapted from Meng et al. [140]) and chemical structures of α-syn fibrillation inhibiting flavonoids.

Flavonoids act as scavenger for free radicals, lowering as such oxidative stress, which has been shown to be relevant in PD pathogenesis due to generation of species such as free radicals and superoxide. For this reason, several flavonoids were assessed for their ability to inhibit α-syn fibrillation in vitro [140]. The assay, based on ThT fluorescence, showed that several of these compounds can inhibit α-syn fibrillation (Figure 27) [140]. Complete α-syn fibrillation inhibition was obtained using 50 µM of compounds **6-HP** and **Baicalein** (Figure 27). Furthermore, significant inhibition was also detected for 7 additional flavonoids: **Eriodictoyl**, **22-324**, **myricetin**, **EGCG**, **T-415**, **22-340/tricetin**, and **22-341** (Figure 27). The flavonoids have not yet been tested in vivo for their ability to image α-syn fibrils. Hence, more comprehensive studies should be conducted [141].

### 3.11. Bicyclic Sulfur-Containing 2-Pyridone Analogues

**Figure 28:**
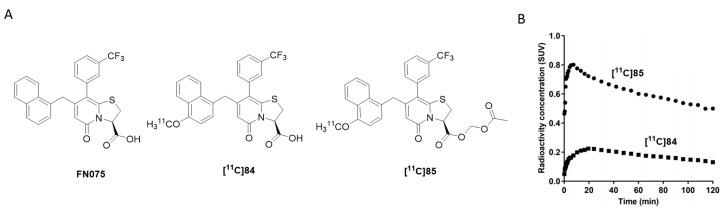
The chemical structures of **FN075** and its analogues. (**A**) The chemical structures of **FN075** and radiolabeled acetoxymethyl ester **[^11^C]84** and **[^11^C]85**. (**B**) TACs in the whole brain derived from healthy NHP following the IV injection of **[^11^C]84** and **[^11^C]85** (reprinted with permission from [146]. Copyright 2018 American Chemical Society).

**FN075** was initially studied as an antibiotic (Figure 28) as it has been shown to inhibit the formation of bacterial amyloid fibers. Interestingly, and contrary to its behavior in bacteria, **FN075** was also identified as a potent α-syn fibril promoter [142]. Prompted by these results, a limited SAR study on **FN075** was conducted. Derivatives altered the lag time of α-syn aggregation in several animal models. Unfortunately, no affinity studies were conducted making it difficult to rationalize the data obtained [143,144,145]. However, the carboxylic acid moiety appeared to be an important feature for the activity since the corresponding esters lost their ability to influence the α-syn fibril formation [142]. In 2018, Cairns et al. developed a first PET tracer based on **FN075**. In order to increase the brain accumulation, they exploited a prodrug approach, masking the carboxylic acid as an acetoxymethyl ester, which was designed to be unstable in vivo and release **FN075** within the brain [143,146]. The authors reasoned that the carboxylic acid moiety disabled the compound’s ability to enter the brain as it becomes deprotonated at physiological pH. A methoxy derivative was also synthesized and evaluated as possible candidate for radiolabeling. In vitro α-syn fibrillization was also triggered by this methoxy analogue **84**, while the acetoxymethyl analogue was inactive. This promising result prompted the group to radiolabel the compound. They reported an A_m_ = > 37 GBq/µmol, an RCP of >97%, but no RCY. **[^11^C]84** (Figure 28A) as well as its protected derivative **[^11^C]85** (Figure 28A) were evaluated in vivo in healthy NHP by dynamic PET imaging (Figure 28B). **[^11^C]85** was able to cross the BBB, showing a slow washed out whereas **[^11^C]84** did not enter the brain (Figure 28B). The authors speculated that **[^11^C]85** was hydrolyzed within the brain however this needs to be proved conclusively [146]. Further studies are needed to determine the potential of **FN075** analogues as possible agents for the imaging of α-syn fibrillization. In particular, affinity and selectivity measurements should be conducted.

**Figure 29:**
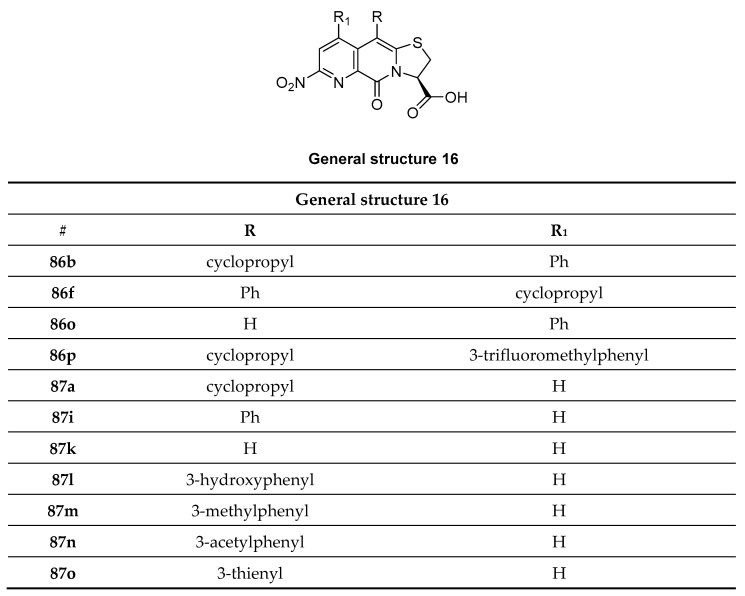
Chemical structures of novel pyridine-fused 2-pyridones (**86b**, **f**, **o**, **p**, **87a**, **i**, **k**–**o**).

Next, Singh et al. synthesized several structural analogues around a tricyclic skeleton (general structure 16, Figure 29) [147]. ThT displacement assays (expressed as relative fluorescence %) on α-syn and Aβ_1–40_ fibrils indicated that p-nitrophenyl derivatives (**86b**, **f**, **o**, **p**, **87a**, **i**, **k**–**o**) displayed affinity toward both fibril types. Other electron-withdrawing groups such as 4-trifluoromethyl and 4-cyanophenyl did not achieve comparable binding results.

**Figure 30:**
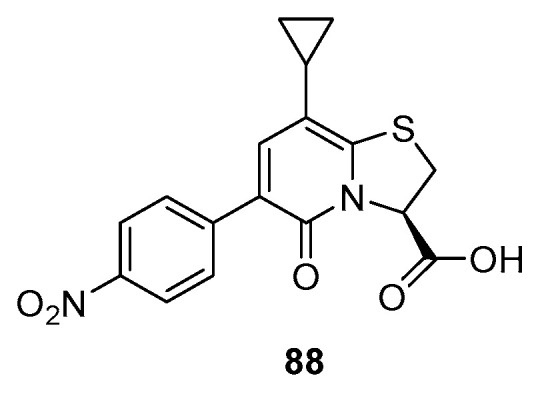
Chemical structure of compound **88**.

A non-pyridine-based fused-peptidomimetic scaffold was also synthesized (**88**, Figure 30), but did not show any significant binding toward α-syn fibrils. This indicates that a pyridine-fused tricyclic backbone in this class of compounds is critical for α-syn fibril binding [147].

### 3.12. N,N-Dibenzylcinnamamide Derivatives

**Figure 31:**
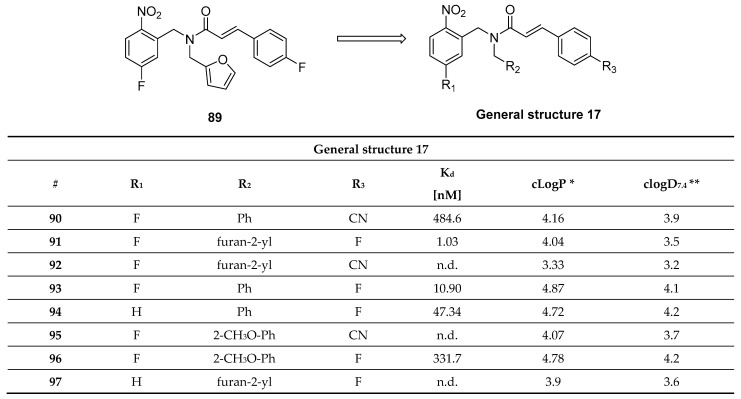
The DBC derivatives tested in SPR assay and structure of DBC compound with binding affinities [148]. * clogP values were calculated by BioByte. ** clogD_7.4_ values calculated by ACD/Percepta.

A recently published study by Chen et al. utilized Surface Plasmon Resonance (SPR) screening to identify *N,N*-dibenzylcinnamamide (DBC) derivatives as high affinity α-syn binders [148]. Among 39 compounds, one hit compound was identified with an adequate K_d_ of 8.21 nM (**89**, Figure 31). Based on these results, eight novel DBC analogues were designed, synthesized, and evaluated against α-syn fibrils. Two compounds (**91** and **93**) showed good binding affinities with K_d_ values of 1.03 nM and 10.90 nM, respectively. These affinity data lead to three hypotheses. Firstly, fluorine as R_1_ substituent appeared to have an essential role for α-syn binding. For example, compound **91** showed significantly better affinity than compound **97**. Secondly, furan-2-yl as the R_2_ substituent increased the affinity substantially in comparison to either phenyl or 2-CH_3_O-phenyl substituents. Thirdly, fluorine as the R_3_ substituent drastically increased binding affinity in comparison to a cyano group [148]. No further evaluation studies have been reported thus far for this compound class, including selectivity and affinity data from post-mortem PD brain tissue on PD. However, this compounds class nonetheless warrants further investigation.

### 3.13. Benzofuranone

**Figure 32:**
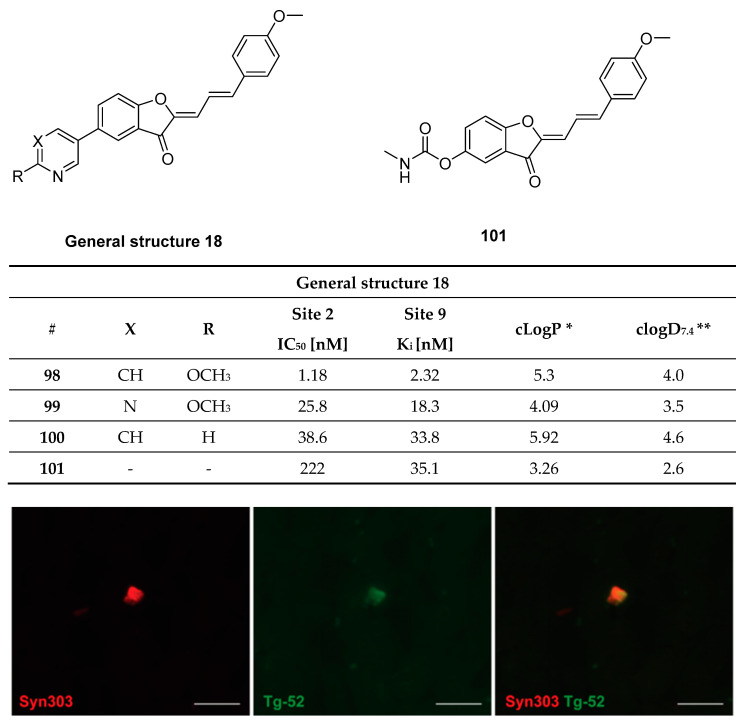
Chemical structure of **99**–**101** with binding IC_50_ and K_i_ values. Fluorescence microscopy studies of **98** in postmortem PD tissue. Image shows LB immunostained with syn303 antibody and **98**.Reprinted with permission from [149]. Copyright 2020 The Royal Society of Chemistry. * clogP values were calculated by BioByte. ** clogD_7.4_ values calculated by ACD/Percepta.

Due their structural similarity with known α-syn ligands such as **15a** (Figure 11), a small set of benzofuranone derivatives (Figure 32) was synthesized and tested for its ability to bind to α-syn fibrils [149]. Compound **98** was identified to be the most promising structure with a K_i_ of 2.32 nM for the α-syn fibril BS9 and an IC_50_ value of 1.18 nM for BS2. These affinity data were determined by a competition binding assay using **[^3^H]Tg-190b** and **[^3^H]BF-2846** (Figure 26A). Encouraged by these results, the binding properties of **98** were evaluated (at 10 µM) on human post-mortem PD, AD, and MSA brain sections. Interestingly, **98** was able to distinguish between LB, LN, and GCI pathology. LB from PD brain sections (cortex) and Aβ plaques from AD brain tissue demonstrated the highest binding. Conversely, a weak binding for of GCI from MSA brain section (cerebellum) and no binding to LN from PD brain sections was obtained (Figure 32) [149]. Presently, no in vivo study has been conducted with these compounds.

### 3.14. Bisquinolines

**Figure 33:**
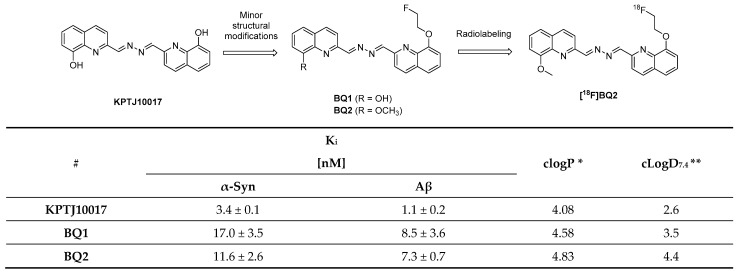
Chemical structure of **KPTJ10017**, **BQ1**, **BQ2**, and **[^18^F]BQ2** with binding affinities (K_i_ values) for recombinant α-syn and Aβ aggregates [120].* clogP values were calculated by BioByte. ** clogD_7.4_ values calculated by ACD/Percepta.

Bisquinolines are a new class of compounds that were identified as potential α-syn ligands just recently [120]. A library of 44.000 compounds was screened for structures with high planarity (to recognize β-sheet structures of amyloids), moderate molecular weight (in order to pass the BBB), and the ability to introduce a radionuclide into the lead structure. Based on these criteria, 150 compounds were selected and further evaluated using an inhibition ThT binding assay. **KPTJ10017** (Figure 33) showed the highest inhibition (62.1%). Next, Kaide and colleagues modified the chemical structure of the lead with the aim of introducing a fluorine atom into the molecule while only minimally changing its physicochemical properties. This led to the identification of **BQ1** and **BQ2** (Figure 33), which showed K_i_ values of 17.0 and 11.6 nM respectively, in a ThT competition assays using recombinant α-syn aggregates. Selectivity of both compounds for α-syn aggregates over Aβ was not optimal and further SAR studies are needed to reduce the ligands affinity to off-target proteins (Figure 33). However, **BQ1** and **BQ2** can detect α-syn aggregates in human PD brain sections using a fluorescent staining-based experiments. **BQ1** and **BQ2** were also able to detect Aβ fibrils of AD patients in brain tissue whereas no binding to tau fibrils was observed. Finally, compound **[^18^F]BQ2** (Figure 33) was radiolabeled (RCY = 1.2%, A_m_ = 8.9 GBq/μmol) and evaluated in vivo in naïve mice. A moderate brain uptake (1.59% ID/g at 2 min) and slow clearance (1.35% ID/g at 60 min) was detected. This retention could be caused by binding to myelin sheaths, monoamine oxidases, or neuromelanins. Finally, ARG studies were conducted on PD brain sections. A high non-specific binding component in the whole region was detected, suggesting that **[^18^F]BQ2** as such is not suitable for α-syn PET imaging [120]. Further SAR studies are therefore needed to improve the affinity and selectivity profile of BQ analogues.

## 4. Conclusions

Considerable research has been devoted, over the past decades, to the identification of α-syn ligands and a variety of chemically diverse compounds have been evaluated. Approximately 37 different chemical classes have been developed and among them 23 compounds were further radiolabeled and evaluated in preclinical studies (Table S2, Supporting Information). Only two compounds (**[^11^C]MODAG-001** and **[^125^I]TZ6184**) showed high affinity (<1 nM) while the rest showed moderate biding toward α-syn fibrils. Four compounds (**[^18^F]2FBox**, **[^18^F]15a**, **[^11^C]MODAG-001** and **[^18^F]S3-1**) showed high selectivity (30–50-fold) over structurally related amyloid proteins as Aβ and tau fibrils. Most importantly, only 12 were evaluated using human derived brain material and among them six showed binding to α-syn. However, these compounds were either unable to selectively bind to α-syn or its binding was tested only by fluorescent staining and not ARG which is considered to be the “gold standard”. In our opinion, the absence of a valid candidate for α-syn PET imaging is mainly related to the lack of reliable and reproducible assays. As a matter of fact, the most challenging aspect over the years has been the evaluation process of these compounds.

Thus far, a variety of in vitro approaches for the determination of binding affinity on fibrils have been described. However, it is unclear if the specific assays used for compound screening are able to accurately mimic the pathophysiology present in human derived tissue. Recently, Schweighauser et al. [150] found structural discrepancies in MSA α-syn filaments in comparison to in vitro “synthetic” filaments (from recombinant wild-type and mutant α-syn). In particular, the size and the packaging of protofilaments seems to differ in human derived tissue compared to “synthetic” fibrils [150]. Moreover, the authors suggested that different strains of α-syn might exist in DLB and MSA patients. Similar hypothesis has been put forward by several other scientists [63,151]. By contrast, Shahnawaz et al., employing tissues derived from MSA and PD patients, demonstrated with cryo-electron tomography that the structure of α-syn fibrils amplified from human material is consistent with the structure of “synthetic” α-syn fibrils prepared in vitro [63]. The discrepancy in results between these two publications is concerning and questions current approaches to design, develop and evaluate α-syn PET tracers. A possible alternative to synthetic fibrils may be sarkosyl-insoluble α-syn material which has been used in more recent studies [150].

Up-to-date, **ThT** competition assays are often used as the only method to evaluate binding, although it has been proved that α-syn fibrils contain multiple binding sites [43]. It might be possible that **ThT** based assays cannot distinguish between compounds that do not bind to the same binding site. Furthermore, many scientists have questioned the reproducibility of these assays, especially given the discrepancies in the reported binding affinities of the studied compounds [67,98,99,114]. The observed discrepancies may be related to several factors, such as, the difference in fibril preparation or practicality of the assay, for example. More reliable and reproducible binding assays are needed in the future to address this challenge. High throughput screening (HTS) may represent an alternative approach to identify α-syn ligands. Until now, only two HTS studies using human derived tissues have been reported [113,114]. Rational drug design is as a matter of fact another alternative development approach. However, this strategy, and in particular structure-based drug design, is hindered as a high resolution crystal structure of α-syn fibril remains elusive. Despite this, several groups have based their research on explorative computational modeling and have identified putative binding sites on α-syn fibrils [43]. We speculate that cryo-electron microscopy (cryo-EM) studies of α-syn from PD, MSA or DLB patients will be a valuable tool in the future that will help to guide the design and development of new imaging probes providing a clearer picture of available binding sites. Finally, a completely different solution to develop α-syn imaging agents is to engineer specific antibodies. A similar approach has been already successfully employed in PET imaging of Aβ [152,153,154].

In conclusion, an α-syn selective PET tracer is still missing and no clear pathway is foreseen to develop such a tracer in the near future. Recent findings studying PD revealed that Lewy pathology is complex and consists of a variety of fragmented membranes, organelles and vesicles [151]. This may explain why the development of α-syn PET tracer has faced several challenges. However, the first promising results have been published and hope is on the rise, as outlined in this review.

## Data Availability

Data sharing not applicable.

## Supplementary Materials

The following are available online at https://www.mdpi.com/article/10.3390/ph14090847/s1, Table S1: Summary of search strategy based on PRISM guidelines, Table S2: Summary table of all radiolabeled compounds with reported results from in vitro assays and preclinical studies.
