# Supplementary material for: Alpha-Synuclein PET Tracer Development—An Overview about Current Efforts
Source: Pharmaceuticals (Basel). 2021 Aug 26;14(9):847. doi: 10.3390/ph14090847 (PMC8466155; doi:10.3390/ph14090847)
Supplement: Supplementary file 1 [file pharmaceuticals-14-00847-s001.zip › pharmaceuticals-1309350-supplementary.pdf]

Supporting Information:

1. Search Strategy

Table S1. Summary of search strategy based on PRISM guidelines.

Section/topic	#	Checklist item	Location(s) Reported	Timing
**INFORMATION SOURCES AND METHODS**				
Database name	1	PubChem	https://pubchem.ncbi.nlm.nih.gov	May 2019 – November 2020
		ChEMBL	https://www.ebi.ac.uk/chembl/	May 2019 – November 2019
		Reaxys	https://www.reaxys.com	May 2019 – November 2019
		World Intellectual Property Organization (WIPO)	https://www.wipo.int/portal/en/index.html	May – July 2019
		PubMed	https://pubmed.ncbi.nlm.nih.gov/	May 2019 – November 2020
Online resources and browsing	2	International Society of Radiopharmaceutical Sciences conference 2019		May 2019 – November 2019
		Annual Congress of the European Association of Nuclear Medicine		May 2019 – November 2019
**SEARCH STRATEGIES**				
Full search strategies	1	PubChem search profile	SNCA gene (human) – sort by activity	-

	2	ChEMBL search profile	Target SNCA – division based on standard units and sort by nM activities	-
	3	Reaxys search profile	Target search (α-syn, human-wild)	-
	4	World Intellectual Property Organization (WIPO) search profile	Search profile: compounds and alpha and synuclein	-
	5	PubMed search profile	Manual search about alpha synuclein and PET imaging	-

2. Summary table of Radiolabeled compounds

Table S2. Summary table of all radiolabeled compounds with reported results from *in vitro* assays and preclinical studies. *Note to the table:* *n.d. = not determined;*

**[#]**	***In vitro* assays**			**Preclinical evaluation**				**Advantages**	**Limitations**
	**Affinity [< 1 nM]**	**Selectivity [30-50 fold]**	**Tested on LB-, LN-, GCI-enriched brain fraction**	**RCY [%]**	**A_M [GBq/µmol]**	**BBB permeability with early peak uptake of SUV > 1.5**	**≥0.4% ID/g (rat brain) or ≥4.0% ID/g (mouse brain)**		
**[^{18}F]SIL26**	No.	No.	Yes	55 – 65 (decay-corrected to EOB)	>200	Passing BBB. No *in vivo* PET imaging.	No.	- moderate affinity on human PD brain homogenates	- low brain uptake and slow clearance
**[^{11}C]SIL5**	No.	No.	Yes.	35 – 45 (decay-corrected to EOB)	>363	Yes.	No.	- high brain uptake	- low affinity on human PD brain homogenates<br>- low selectivity profile

**[^{11}C]BF-227**	No.	No.	Yes.	>50% (based on [^{11}C]CH_3OTf)	119-138	Yes.	N.d.	- confirmed binding to GCI rich brain regions confirmed by PET study on patients	- contradictory results among different research groups concerning binding towards α-syn aggregates
**[^{18}F]BF-227**	No.	No.	Yes.	N.d.	40-840	N.d.	N.d.	-	- no binding towards α-syn aggregates.
**[^{18}F]2FBox**	No.	Yes.	Yes.	10 – 19	68-543	Passing BBB, SUV < 1.5	N.d.	-	-no binding to α-syn aggregates confirmed by postmortem tissue investigation.
**[^{18}F]15a**	No.	Yes.	No.	N.d.	29.6-185	N.d.	N.d.	- high binding affinity and selectivity.	- too lipophilic, slow clearance<br>- lack of information about binding in human derived brain tissue
**[^{18}F]S3-1**	No.	Yes.	N.d.	N.d.	N.d.	No.	No.	- high affinity and selectivity	- lack of information about biding in human derived brain tissue<br>- lack of more detailed scientific literature<br>- rapid clearance with marginally increase in the later timepoints
**[^{11}C]PBB3**	No.	No.	Yes.	N.d.	133	-	-	-moderate affinity tested on human derived brain tissue (DLB)	- low selectivity
**[^{3}H]C05-01**	No.	No.	Yes.	N.d.	0.81	N.d.	N.d.	-high affinity tested on human derived DLB brain tissue	-low selectivity
**[^{125}I]BI-2**	No.	No.	Yes.	N.d.	81.4	Yes.	No.	- moderate selectivity	- low affinity<br>- high lipophilicity and low brain uptake
**[^{11}C]anle253b**	N.d.	N.d.	No.	47	15.1±3.4	-	Yes.	-IC_{50} value of 1.6 nM	-lack of information about affinity using human derived brain tissue<br>-moderate brain uptake

**[^{11}C]MODAG-001**	Yes.	Yes.	Yes.	3.6 ± 1.1 and 11.4 ± 3.7	31.3 ± 6.4 and 98.6 ± 24.7	Yes.	Yes.	- high affinity<br><br>- no binding to aggregated tau was detected in PSP and AD brain tissues	-fast metabolism and detection of radiometabolites in the mouse brain<br><br>- lack of strong binding and high nonspecific binding in human LBD brain tissue
**[^{125}I]IDP-4**	No.	No.	Yes.	19-60	81.4	Passing BBB.	Yes.	-high binding affinity and moderate selectivity	- low brain uptake
**[^{18}F]42**	No.	No.	No.	32 ± 3	128	Passing BBB.	N.d.	-high affinity<br><br>-moderate affinity only over Aβ	-low selectivity over tau<br><br>-lack of information about binding using human derived brain tissue<br><br>- lack of detailed scientific literature
**[^{11}C]52**	No.	No.	No.	50±10	>148 (d.c. to EOB)	N.d.	N.d.	-	-lack of selectivity
**[^{18}F]53**	No.	No.	No.	21 ± 7	>27 (d.c. to EOS)	N.d.	N.d.	- moderate affinity towards α-syn fibrils	-lack of selectivity
**[^{18}F]54**	No.	No.	No.	30 ± 4	>37 (d.c. to EOS)	N.d.	N.d.	- moderate affinity towards α-syn fibrils	-lack of selectivity
**[^{125}I]TZ6184**	Yes.	N.d.	N.d.	50-60	N.d.	N.d.	N.d.	-excellent binding affinity on α-syn fibrils	-lack of information about selectivity profile<br><br>-limited detailed scientific literature available
**[^{125}I]83**	No.	No.	No.	57	81	N.d.	N.d.	-high binding affinity<br><br>-moderate selectivity	-lack of binding affinity tested on human derived tissue<br><br>-high non-specific binding
**[^{3}H]55**	N.d.	N.d.	Yes.	N.d.	77 Ci/mmol	-	-	-	-lack of detailed scientific literature

**[^{11}C]84**	N.d.	N.d.	No.	N.d	>37	Passing BBB. Evaluated in NHP.	-	-	-lack of binding and selectivity profile tested on human derived brain tissue
**[^{11}C]85**	N.d.	N.d.	No.	N.d.	>37	Passing BBB. Evaluated in NHP.	-	-	
**[^{18}F]BQ2**	No.	No.	Yes.	1.2	8.9	N.d.	No.	-moderate brain uptake and binding affinity	-lack of selectivity<br>-high non-specific binding-
